# RiceProteomeDB (RPDB): a user-friendly database for proteomics data storage, retrieval, and analysis

**DOI:** 10.1038/s41598-024-54151-4

**Published:** 2024-02-14

**Authors:** Dong U Woo, Yejin Lee, Cheol Woo Min, Sun Tae Kim, Yang Jae Kang

**Affiliations:** 1 Division of Bio & Medical Bigdata Department (BK4 Program), Gyeongsang National University, 501, Jinju-daero, Jinju-si, Gyeongsangnam-do 52828 Republic of Korea; 2 Department of Plant Bioscience, Life and Industry Convergence Research Institute, Pusan National University, Milyang, 50463 Republic of Korea; 3 Division of Life Science Department, Gyeongsang National University, Jinju, 52828 Republic of Korea

**Keywords:** Proteomics, Bioinformatics

## Abstract

Rice, feeding a significant portion of the world, poses unique proteomic challenges critical to agricultural research and global food security. The complexity of the rice proteome, influenced by various genetic and environmental factors, demands specialized analytical approaches for effective study. The central challenges in rice proteomics lie in developing custom methods suited to the unique aspects of rice biology. These include data preprocessing, method selection, and result validation, all of which are essential for advancing rice research. Our aim is to decode these proteomic intricacies to facilitate breakthroughs in strain improvement, disease resistance, and yield optimization, all vital for combating global food insecurity. To achieve this, we have created the RiceProteomeDB (RPDB), a React + Django database, offering a streamlined and comprehensive platform for the analysis of rice proteomics data. RiceProteomeDB (RPDB) simplifies proteomics data management and analysis. It offers features for data organization, preprocessing, method selection, result validation, and data sharing. Researchers can access processed rice proteomics data, conduct analyses, and explore experimental conditions. The user-friendly web interface enhances navigation and interaction. RPDB fosters collaboration by enabling data sharing and proper acknowledgment of sources, contributing to proteomics research and knowledge dissemination. Availability and implementation: Web application: http://riceproteome.plantprofile.net/. The web application’s source code, user’s manual, and sample data: https://github.com/dongu7610/Riceproteome.

## Introduction

Over the past two decades, proteome data production and the efficiency of its analysis have seen an exponential increase, leading to the accumulation of data in the PRIDE (Proteomics Identification Database,^1^). Scientists are currently attempting to construct a proteome big data set in order to gain a wider perspective on proteome expression. To save time, bioinformaticians are developing automated pipelines, that can integrate proteome data into larger matrices, enabling new discoveries. This process involves multiple steps, such as data processing, quantification, analysis, and storage and sharing. The data processing and quantification step is the most computationally intensive, and the software Maxquant^2^ is commonly used. However, it can be difficult for scientists without bioinformatics knowledge to translate complex output formats from Maxquant for large-scale analysis in Linux environments so as to automate the process and compare various proteome studies.

Furthermore, in order to minimize any potential biases in the analysis, it is important to apply appropriate and comparable statistical techniques to the data. Following the removal of contaminants, the imputation of missing values is addressed using various methods^3^. This includes employing random forest (RF) imputation through the R package MissForest^4^, principal component analysis (PCA) via the R package missMDA^5^, and k-nearest neighbors (KNN) imputation using the R package VIM^6^. Additionally, the choice of proteomics experimental method will dictate the appropriate normalization strategy. In the case of Label-Free Quantification (LFQ^7^), which allows for the quantification of proteins without labeling and utilizes the relative abundance of peptides to compare protein expression levels across different samples, normalization can be performed using either quantile normalization or variance stabilization normalization (VSN) using the R package vsn^8^. On the other hand, tandem mass tag (TMT)^9^ labeling requires more accurate quantification of proteins by measuring the relative abundance of labeled peptides. To ensure consistency in results across samples, internal reference scaling (IRS) normalization^10^ is necessary for TMT quantification. Furthermore, to ensure effective and efficient analysis of proteomics data, it is critical to store quantification results in a structured manner.

In this study, we have developed a web-based proteomics data analysis pipeline specifically tailored for the annotation of rice proteins (*Oryza sativa*). The pipeline begins with the processing of Maxquant output files and continues through data pre-processing, analysis, and visualization, providing a comprehensive and efficient approach for analyzing proteomic data. Additionally, for LFQ studies, we have enhanced our pipeline by integrating FragPipe, which allows for the analysis of the ‘combined_protein’ output file. This pipeline is developed to empower researchers to effectively manage, analyze, and interpret their data by React framework for creating user-interface and Django-Celery for executing the pipeline asynchronously and efficiently. The pipeline is user-friendly and accessible to non-bioinformaticians and is also capable of handling large datasets. The functional component to create secondary analytic branches for the re-analysis facilitates the management of custom revisits to the data and the sharing of tailored results. The processed data can be analyzed using statistical and bioinformatics methods such as T-test, Gene Ontology (GO), and network analysis, and can be visualized using the google charts API. The web-based platform, RPDB, is expected to be a useful tool and resource for researchers studying the proteome of *O. sativa*.

While the platforms amica^11^ and LFQ-Analyst^12^ are known for their user-friendly analysis tools, our platform stands out by enabling researchers to upload their data. This data is then converted into a format that is publicly accessible, offering a significant advantage for broader data sharing and collaboration. This feature fosters a more collaborative environment and supports the principles of open science, going beyond just ease of use. By facilitating access to shared data, our platform positions itself as a vital resource in rice proteomics research, encouraging a more inclusive and collaborative approach among the scientific community.

## Materials and methods

### Data collection and preparation

Proteomics data used in this study were obtained from experiments conducted on *O. sativa*. For the analysis, both the proteingroups output file and the Experiment design file were utilized as input. The proteingroups file, generated through the Maxquant software, contains the quantified protein expression levels. Concurrently, the Experiment design file provides essential context for the quantitative data, summarizing the experimental setup with key information such as Experiment Name, Sample Name, Condition, and Replicate. These elements together offer a comprehensive overview of the protein groups identified in the experiment, allowing for a more informed and contextualized analysis. The structured presentation and organization of both the proteingroups data and the experiment design are instrumental in correlating the quantitative proteomic data with the biological context. To further aid understanding, the Supplementary Data included with this document presents a detailed perspective on the sample data and experimental design, proving invaluable for appreciating the structure and background of the proteomic analysis in our research. In this study, we utilized proteomic data, specifically employing the PRIDE ID: PXD008069. The data analysis was conducted using the label-free quantification (LFQ) approach. For the protein identification and quantification, we utilized MaxQuant software (version 1.5.3.12). This choice was guided by its efficiency in handling large-scale proteomic data and its wide acceptance in the scientific community for proteomic analyses. Additionally, another set of proteingroups data related to different samples has been provided in the Supplementary Information.

### Data preprocessing

Preprocessing methods varied depending on the quantification approach. For label-free quantification (LFQ), which does not require labeling, protein expression levels were compared across different samples using the relative abundance of peptides. The preprocessing steps for LFQ, which were influenced by the methods described in references^13,14^, included contaminant removal, normalization, and imputation. For normalization step, both quantile normalization and VSN are considered. In the imputation step, a range of methods can be employed, including RF (ntree = 100), PCA (ncp = 3), and KNN (k = 5).

For tandem mass tag (TMT) labeling, a different quantification approach was used, involving precise quantification of proteins by measuring the relative abundance of labeled peptides. The preprocessing steps for TMT, which were influenced by the methods described in references^3,10^ and the GitHub repository (https://pwilmart.github.io/blog/2018/12/12/TMT-zero-replacement), included contaminant removal, imputation, and IRS normalization. For the imputation step, a choice can be made among various methods such as RF (ntree = 100), PCA (ncp = 3), KNN (k = 5). Furthermore, the rpy2 package (https://pypi.python.org/pypi/rpy2) was utilized to write R scripts within the Django framework.

### Statistical analysis

To identify differentially expressed proteins (DEP), a statistical analysis was conducted. The t-test was used to determine significant differences in protein expression levels between conditions. The statistical analysis was performed using Python and the SciPy library^15^.

### Gene Ontology (GO) analysis

To gain insights into the functional annotations of the identified proteins, Gene Ontology (GO) analysis was conducted using the goatools package, a Python library for accessing and analyzing GO annotations^16^. The analysis involved mapping the identified proteins to GO terms and determining enriched terms. For this analysis, the japonica type data from ftp.ebi.ac.uk/pub/databases/GO/goa/proteomes/2610640.O_sativa_subsp_japonica_Rice.goa was used as the reference dataset^17^. This reference dataset provides specific information about the GO annotations of proteins in the japonica type of *O. sativa* (rice). By comparing the identified proteins with the GO annotations in this reference dataset, enriched GO terms associated with the identified proteins were determined, providing valuable insights into their functional annotations.

### Network analysis

The RiceNet database served as the network source for protein interaction analysis, providing the necessary data^18^. The NetworkX library, a powerful Python library for network analysis and manipulation, was utilized to construct the two-step-neighbor network^19^. This network representation enabled the analysis of protein interaction patterns and identification of key network components. The utilization of the RiceNet database and NetworkX library provided valuable resources and tools for exploring the protein network and understanding protein relationships.

### Web-based platform

A web-based platform was developed using Django (https://www.djangoproject.com/) and Django-Celery (http://www.celeryproject.org/) to efficiently manage workflows and execute tasks asynchronously. The pipeline was containerized with Docker, providing scalability and portability. The frontend was built using React (https://react.dev/) to create a user-friendly interface, enabling seamless interaction with the pipeline. For visualization purposes, data was received through a REST API in React. Interactive charts and networks were generated using React Google Charts (https://www.react-google-charts.com/) and React Force Graph (https://github.com/vasturiano/react-force-graph). CSS frameworks like React Bootstrap (https://react-bootstrap.netlify.app/) and React Semantic UI (https://react.semantic-ui.com/) were integrated to enhance the visual appearance and styling of the user interface.

The web-based platform, RPDB, empowered researchers to effectively manage, analyze, and interpret their proteomics data. It facilitated the processing of Maxquant output files, data pre-processing, statistical analysis, GO analysis, and network analysis. The platform was accessible to non-bioinformaticians, enabling easy data exploration and sharing of tailored results.

## Results

### Overall design of user-friendly platform for analyzing and storing the proteome data

In order to enhance the accessibility of proteome data analysis, we developed a web application utilizing React, Django, and Celery. This application allows for the upload of data, which then undergoes pre-processing and is stored in a database. Furthermore, the application allows users to select previously uploaded experiments and perform a step-by-step analysis, including differentially expressed proteins (DEP), gene ontology (GO), and network analysis. Once a project is created, it can be selected and accessed to upload experiments (MaxQuant output data: “proteingroups”). The uploaded experiments can then be selected, and various analyses can be performed by creating the branches for the desired analysis.

The analysis process consists of the following steps: (1) Differentially expressed proteins (DEP) analysis involves assigning the conditions to be compared, identifying significant proteins through a T-test, and visualizing the results using volcano plots, manhattan plots, and venn diagrams. (2) GO analysis is the next step, where the significant proteins identified through DEP analysis are subjected to Gene Ontology Enrichment Analysis (GOEA). (3) The gene selection step is the preparation stage for the network analysis. The proteins considered significant from both the DEP and GO analysis can be added to a “Protein basket” for use in the network analysis. (4) In the network analysis phase, the user can choose a protein from the “Protein basket” to display its 2-step neighbor network. Upon completion of the analysis, the branch can be saved, enabling the user to review the results along with metadata (Supplementary Fig. S1).

The series of processes described in the flowchart (Supplementary Fig. S1) is performed within the web application. These requests are received and processed by Django, and can be divided into simple requests (e.g. login, project creation, branch creation, public switching, and etc.) and heavy requests (e.g. data upload, DEP, GO and NETWORK analysis). To avoid reducing the performance of the web page, Celery handles big requests while Django handled basic requests. The results of analyses that are stored in the database interact with the web interface using REST API. The user can visualize the results of each process through a React-based UI, which includes tables, volcano plots, manhattan plots, venn diagrams, bar charts, pie charts, and networks. The user interface (UI) was structured to include Project, Experiment, Analysis branch pages.

### Web application features

#### Web application features: project creation and data processing

**Figure 1:**
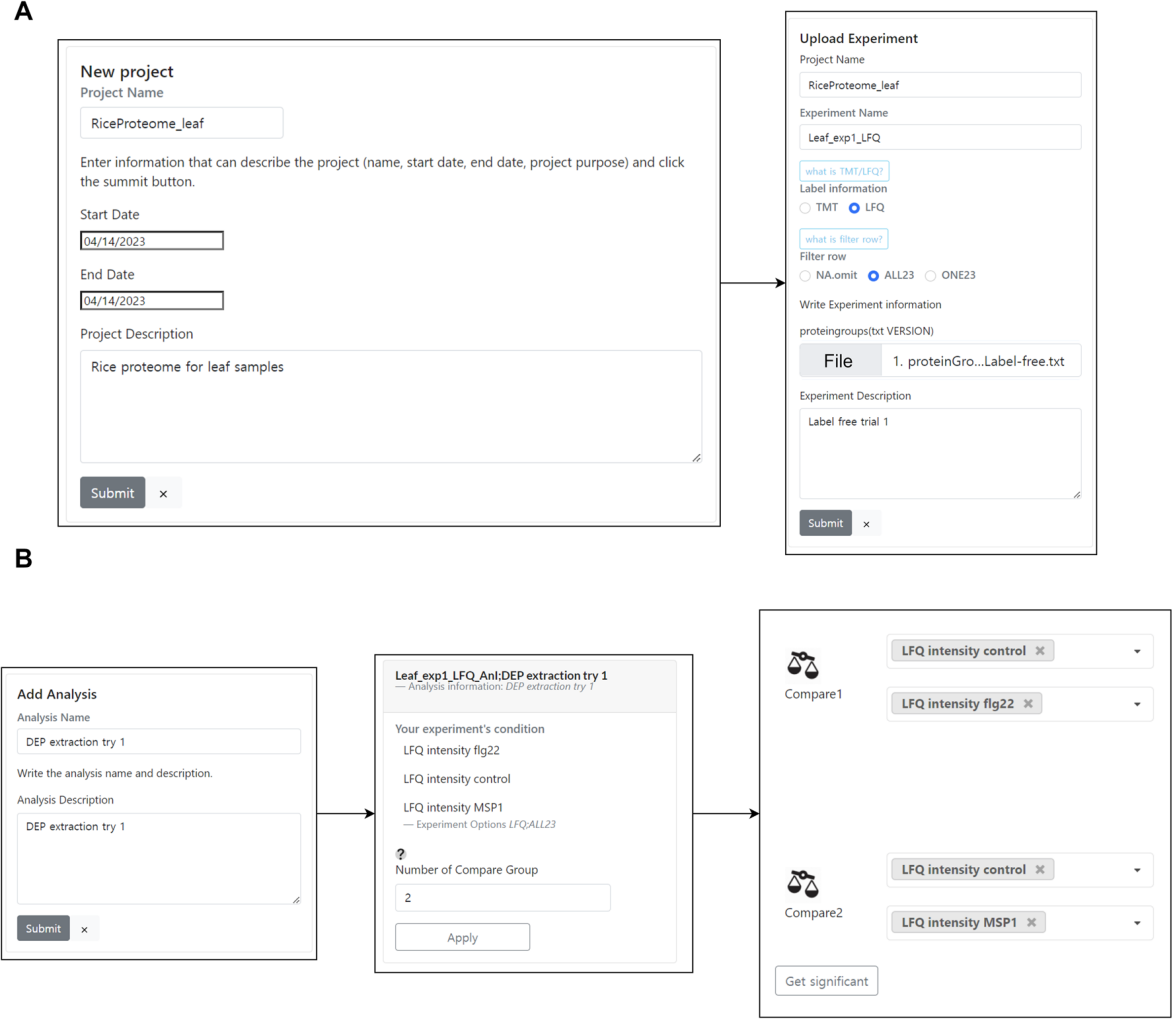
Project creation and analysis workflow. (**A**) The project creation interface allows researchers to register experiments and upload the “proteingroups” file from MaxQuant output. (**B**) An analytic branch can be created for each selected experiment. This functionality allows a number of comparisons for the experimental groups.

The project creation interface facilitates the registration of associated experiments. Within the experiment registration page for each project, researchers are allowed to upload one of the Maxquant output files, specifically the “proteingroups” file. Subsequently, any missing data within the file is systematically handled and normalized according to both LFQ and TMT techniques, which are then recorded within the database. The recorded experiments can be selected for analysis on the experiment page (Fig. 1A). When selecting an experiment to analyze, an analytic branch can be created to proceed with various analyses. The researcher can implement the analyses; differential expression proteomics (DEP), Gene Ontology (GO), and network analysis by selecting the branch. Furthermore, when the experiment is made publicly available with a simple click, other researchers can access the completed analysis and generate new branches for re-analysis in relation to their specific research interests (Fig. 1B).

#### Web application features: analytic branch

**Figure 2:**
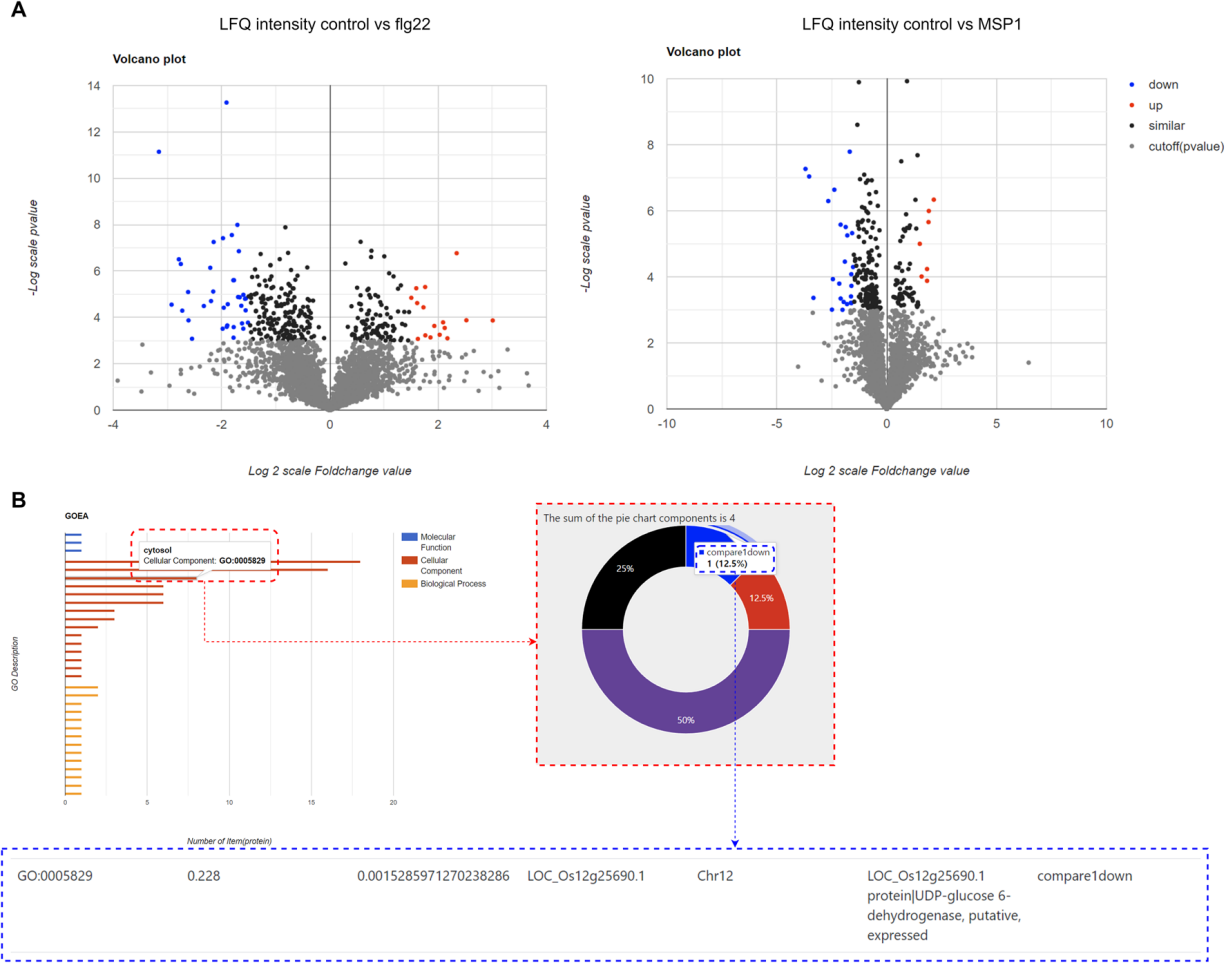
DEP and GO analysis workflow. (**A**) Volcano plots after DEP analysis workflow. (**B**) GO analysis workflow. GOEA results are visualized into GO bar charts, pie charts, and tables with annotations.

When proceeding with the analysis, researchers select comparison groups for the DEP analysis (Fig. 2A). The Python library, SciPy, is employed through the Django framework to perform t-tests, and the results are visualized on a web application using volcano plots, manhattan plots, and venn diagrams. To generate volcano plots for each comparison group, the proteins are assigned colors based on the default thresholds of p-value (− Log[p value] ≥ 3) and fold change. Specifically, up-regulated proteins (Log2[foldChange] ≥ 1.5) are denoted in red, unchanged proteins (− 1.5 < Log2[foldChange] < 1.5) in black, down-regulated proteins (Log2[foldChange] < − 1.5) in blue, and proteins that fall under the cut-off criteria are represented in gray. The proteins that surpass the cut-off for p-value and fold change in each comparison group can be transferred to GO analysis. The list of identified protein list with the *p-*value and fold change can be downloaded in bulk (Supplementary Table S1).

The Python library, goatools, was employed through the Django framework to perform Gene Ontology Enrichment Analysis (GOEA). The results of the GOEA were visualized on a web application using GO bar charts, which can be divided by the major classification criteria of GO (Biological process; BP, Cellular component; CC, Molecular function; MF) and viewed in different colors (Fig. 2B). By selecting a specific bar (GO ID) on the graph, information about proteins included in the GO ID can be displayed in tables and pie charts. The entire GO annotation for the identified proteins can be downloaded in bulk (Supplementary Table S2).

#### Web application features: network analysis using “Protein basket”

**Figure 3:**
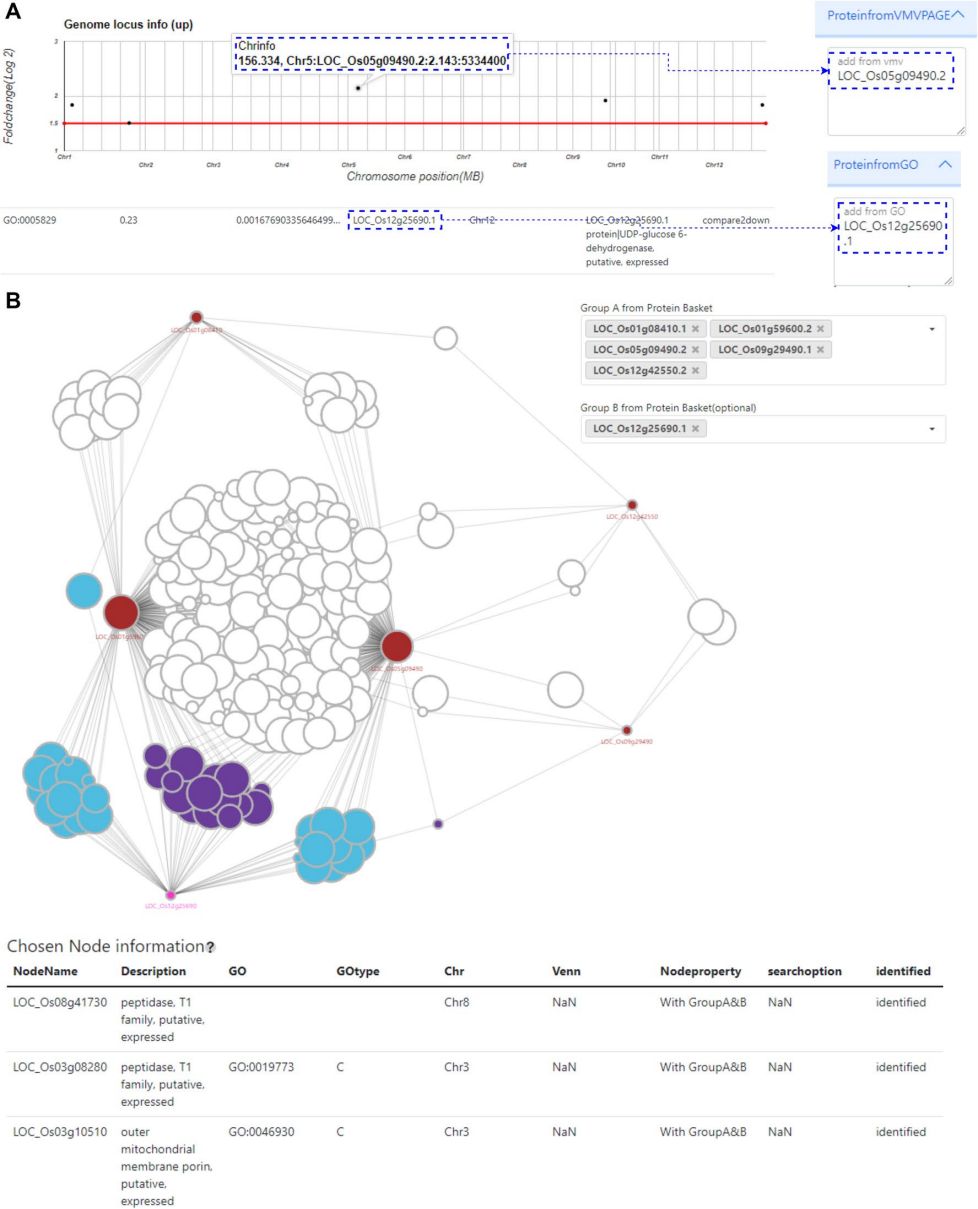
Network analysis and visualization of important proteins. (**A**) Adding proteins of interest: For network analysis, proteins from DEP and GO results can be added to the “Protein basket” by researchers. (**B**) Visualization of proteins from the protein basket, with nodes colored by group membership (groupA or groupB). Selected nodes are displayed in a separate table.

Researchers can add proteins that they consider important from the gene list in DEP and GO results to the “Protein basket”. In addition, known proteins from previous studies can also be compared to proteins identified in the experiment by adding them to the “Protein basket” in bulk (Fig. 3A).

Network analysis can currently be performed on a limited number of important proteins (A group: 10 or fewer) that have been included in the “Protein basket”. This is due to computational performance limitations. The analysis is conducted using the Python library NetworkX and RiceNet, a gene network for rice, and is visualized using 2-step neighbors. It is also possible to include up to 2 additional important proteins (B group: 2 or fewer) for comparison with the previously known candidate genes.

The network diagram visually represents proteins from two distinct groups, A and B, as colored circles. Additionally, the Venn diagram, Gene Ontology (GO), and Chromosome information associate proteins with different colors on the periphery of the circles, depending on the mode of selection; "Default," "Venn," "GO," and "Chr". When a node is displayed, it can be clicked to reveal additional information about the gene. Upon clicking, the node transforms into an orange-colored circle, and its annotations are listed in the "Chosen Node Information" table.

The network analysis results can be downloaded in CSV and Cytoscape formats, allowing for the protein interactions to be further analyzed and visualized by re-drawing the network in Cytoscape. After the analysis is completed, it can be also reviewed again in the "Finished Analysis" section of the "Analysis list" page. This allows for easy access and reference to previously completed analyses.

### Case study: MSP1 and Flg22 with LFQ

#### Data preprocessing

The data obtained from the MaxQuant quantification analysis of samples^20–22^ has been uploaded for further investigation into the defensive response of MSP1 against PTI activation^20^. MSP1 is an effector protein that is secreted by *Magnaporthe oryzae*, a fungus responsible for causing rice blast disease. This protein plays a crucial role during the early stages of the infection by triggering cell death and activating the plant's immune system. By analyzing the proteome data using RPDB, we can obtain a comprehensive understanding of how rice responds to the protein produced by fungus. We have designated the project name as “RICE(PlantDiseaseResistance)”, which is publicly available under the “Experiments (Public)” menu in RPDB.

#### Analysis

From the Venn diagram comparing Group 1 (Control vs MSP1) and Group 2 (Control vs Flg22), we identified upregulated proteins (LOC_Os01g08410.1, LOC_Os01g59600.2, LOC_Os05g09490.2, LOC_Os09g29490.1, LOC_Os12g42550.2) responsive only to MSP1 (Supplementary Table S3). To perform network analysis, we constructed Group A using these proteins. Moreover, we placed our focus on a single cytosolic protein, which was down-regulated in response to MSP1, identified in the GOEA bar chart (Supplementary Table S4). It is a protein denoted as LOC_Os12g25690 that is responsible for encoding UDP-glucose 6-dehydrogenase. The upregulation of two orthologs of peptidase T1, a peroxidase precursor, a methyl-CpG binding domain containing protein, and early fruit mRNA suggests a potential response to MSP1 over-expression. Peptidase T1 and peroxidase precursor may be involved in the plant's defense mechanism, potentially by degrading the effector protein or combating oxidative stress induced by the infection^23,24^. The upregulation of the methyl-CpG binding domain containing protein could indicate alterations in DNA methylation status, a common response to stress or infection in plants^25^ Additionally, the upregulation of early fruit mRNA may signify a shift in plant development or a stress-induced response. Conversely, the downregulation of UDP-glucose 6-dehydrogenase, an enzyme critical for the formation of polysaccharides and glycoproteins in the cell wall, could weaken the cell wall, facilitating the penetration and spread of the fungus^26^.

To conduct network analysis based on RiceNet, we formed two distinct groups. Group A included five upregulated proteins that are associated with MSP1, whereas Group B consisted of the down-regulated cytosolic protein (Fig. 2B). The gene network revealed potential associations between genes in Group A and Group B. However, despite the dense presence of purple-colored genes (Fig. 3B) in the network, no differential expression was observed among them in the dataset.

Using the wholenode.csv file that can be downloaded network analysis page, we used UniProt Citation^27^ to see if the identified proteins were related to stress response (Supplementary Table S5). Through UniProt ID mapping, we found 104 proteins related to Group A, of which 24 were found in stress-related studies. In addition, we confirmed 128 proteins in Group A&B and Group B, and found that 31 proteins (8 of which were duplicates) were reported in stress-related studies.

Since a two-step neighbor network was drawn for each protein, some of the proteins found between the proteins may be stress-related proteins that have not yet been reported in stress-related studies or proteins that have not been mapped to UniProt IDs. In this way, researchers can explore new questions or answers related to stress in these relationships.

## Discussion

RiceProteomeDB (RPDB) is a web-based application and an extensive database that serves as a valuable resource for researchers engaged in rice proteomics research. The application provides researchers with the capability to upload and integrate their output results obtained from MaxQuant, a popular software tool for processing newly generated protein data, as well as data obtained from open-source databases like PRIDE. Researchers can use RPDB to analyze and determine the number of statistically significant proteins found in their submitted data. To aid researchers in selecting the proteins that are pertinent to their research interests, RPDB offers visualization tools like volcano plots, manhattan plots, and venn diagrams. Gene ontology (GO) analysis can be performed using RPDB by creating GOEA bar charts to identify proteins that fall under particular GO IDs. Additionally, network analysis can be carried out by visualizing the interactions between important proteins identified in the previous steps. Researchers can choose to make their experiments public, allowing other researchers to view and re-analyze the data based on their research interests.

To demonstrate how RPDB can be used to acquire insights into the defensive response of MSP1 against PTI activation using MaxQuant outputs, we conducted a case study using previously published data^20^. This study showcases how users can create and manage their projects and easily share them with others. Furthermore, the case study demonstrates how RPDB can offer novel insights into stress-related proteins and their interactions. The discovery of possible regulatory networks highlights the potential of RPDB in uncovering novel regulatory mechanisms from proteome data. Identification of such regulatory factors could lead to the development of stress-tolerant crops, improving global food security.

In summary, our results demonstrate that RPDB is a vital tool for researchers in the field of rice proteomics. It effectively addresses the challenges facing the analysis, visualization, and sharing of massive proteomics data, providing researchers with a user-friendly interactive platform. Future updates of RPDB could include integration with other omics data, making it a more comprehensive data management tool for the entire rice research community.

### Supplementary Information

Supplementary Information 1.

Supplementary Figure 1.

Supplementary Information 3.

Supplementary Information 4.

Supplementary Table 1.

Supplementary Table 2.

Supplementary Table 3.

Supplementary Table 4.

Supplementary Table 5.

## Data Availability

The data used in this study, as well as the source code for the proteomics data analysis pipeline, can be found at the following GitHub repository: https://github.com/dongu7610/Riceproteome. Researchers are encouraged to explore the repository to access the data, review the analysis pipeline, and contribute to the ongoing research in the field of rice proteomics. To help utilize the RiceProteomeDB web application, a comprehensive user manual is provided within Supplementary Manual. This manual offers guidelines for researchers on how to use the web application upon their initial access.
