# Supplementary figures and images for: RiceProteomeDB (RPDB): a user-friendly database for proteomics data storage, retrieval, and analysis
Source: Sci Rep. 2024 Feb 14;14:3671. doi: 10.1038/s41598-024-54151-4 (PMC10864295; doi:10.1038/s41598-024-54151-4)

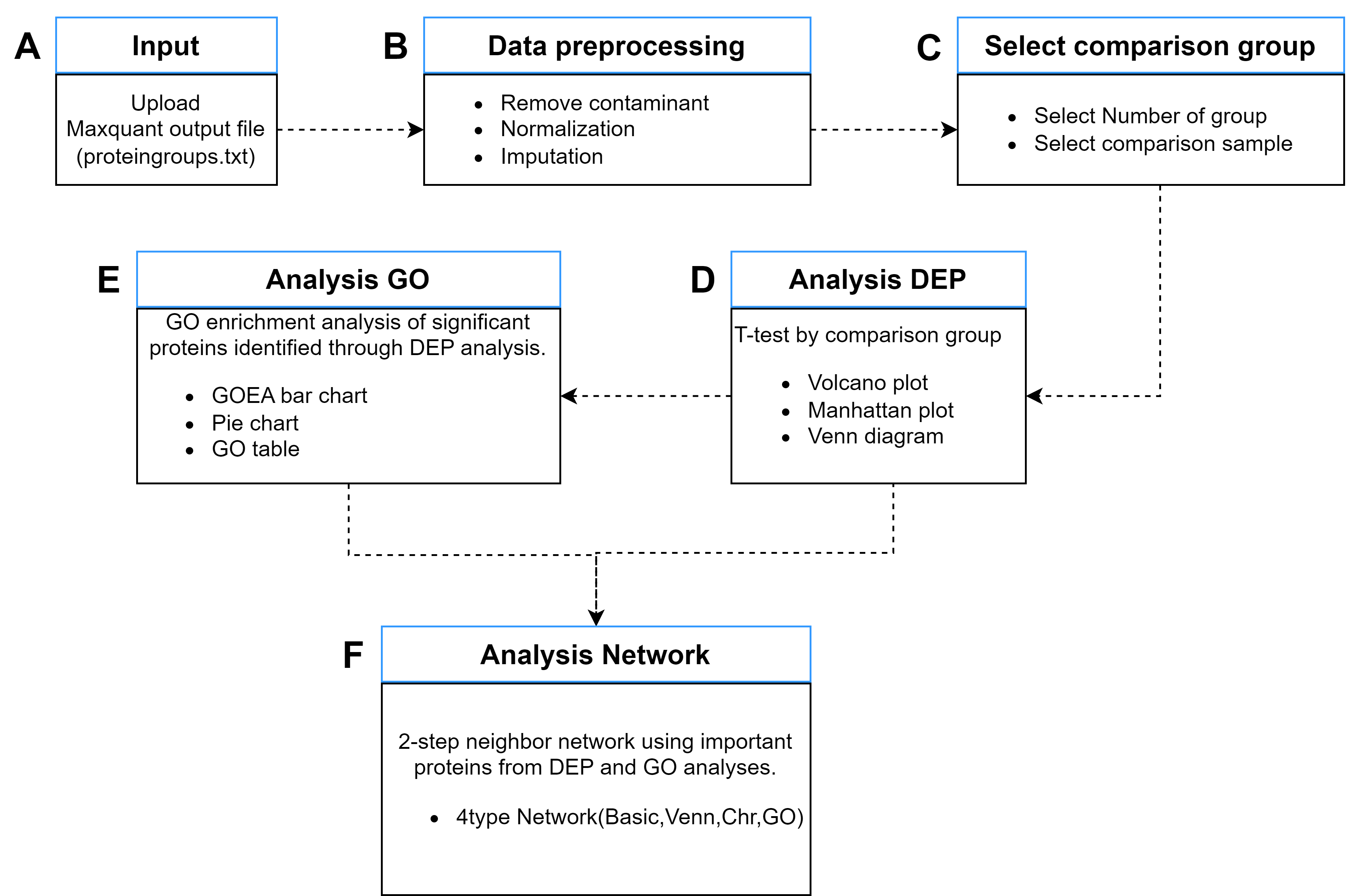

Supplement: Supplementary file 2 — Supplementary Figure 1. [file 41598_2024_54151_MOESM2_ESM.png]
