# Supplementary material for: RiceProteomeDB (RPDB): a user-friendly database for proteomics data storage, retrieval, and analysis
Source: Sci Rep. 2024 Feb 14;14:3671. doi: 10.1038/s41598-024-54151-4 (PMC10864295; doi:10.1038/s41598-024-54151-4)
Supplement: Supplementary file 4 — Supplementary Information 4. [file 41598_2024_54151_MOESM4_ESM.pdf]

User’s manual

RiceProteomeDB

Content

1. INTRODUCTION

1.1. Web application Overview 4
1.2. Organization Manual 5
1.3. Sample Prepare

2. START

2.1. Account 8
2.2. Management
2.2.1. Create Project 9
2.2.1.1. Join Project 12
2.2.2. Upload Experiment 13
2.2.3. Add Analysis 17
2.3. Ongoing Analysis
2.3.1. Analysis DEP 21
2.3.2. Analysis GO 30
2.3.3. Protein Basket 34
2.3.4. Analysis NETWORK 36
2.4. Finish Analysis 44

3. CONTACT

INTRODUCTION

1.1 Web application Overview

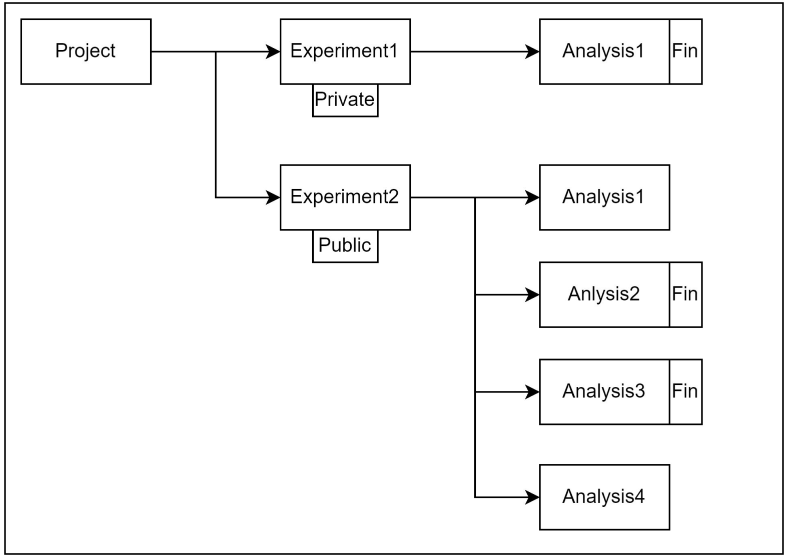

Figure 1. Overview of when users use RiceProteomeDB

- Users can create projects, upload experiments within those projects, and perform multiple analyses on the experiments. Among these, they can change the property of an experiment from 'Private' to 'Public' (Figure 1).
- At this point, users not involved in the project can access finished analyses of public experiments within the 'Experiment (Public)' section (Figure 2).

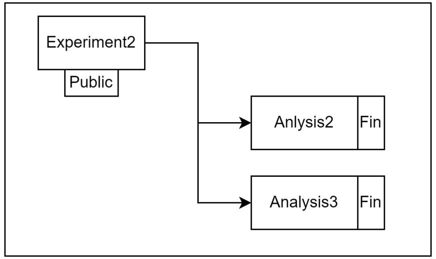

Figure 2. Cases of Users Not Participating in the Project

1.2 Organization Manual

The user manual is structured into sections for Account, Management, Ongoing Analysis, and Finish Analysis

1. **Account Section**: This section provides information on account and login.
2. **Management**: Within this section, users will find comprehensive information about project creation, participation, experiment uploads, and the addition of analyses.
3. **Ongoing Analysis**: The Ongoing Analysis section guides users through sequential DEP, GO, and NETWORK analyses. It also explains how to use the 'Protein Basket' feature, allowing users to add proteins of interest during ongoing analysis.
4. **Finish Analysis**: This section outlines the finalization of the analysis process, including accessing completed analyses and downloading associated files.

In addition, detailed terminology and explanations can be found in the GitHub Wiki section for reference.

1.3 Sample Prepare

Part to be used as value in "proteingroups" file

LFQ intensity control rep1	LFQ intensity control rep2	LFQ intensity control rep3	LFQ intensity flg22 rep1	LFQ intensity flg22 rep2	LFQ intensity flg22 rep3	LFQ intensity MSP1 rep1	LFQ intensity MSP1 rep2	LFQ intensity MSP1 rep3

experimentname	samplename	condition	replicate
LFQ intensity control rep1	control_1	control	1
LFQ intensity control rep2	control_1	control	2
LFQ intensity control rep3	control_1	control	3
LFQ intensity flg22 rep1	flg22_1	flg22	1
LFQ intensity flg22 rep2	flg22_1	flg22	2
LFQ intensity flg22 rep3	flg22_1	flg22	3
LFQ intensity MSP1 rep1	MSP1_1	msp1	1
LFQ intensity MSP1 rep2	MSP1_1	msp1	2
LFQ intensity MSP1 rep3	MSP1_1	msp1	3

- Write down the experiment name, sample name, condition, and replicate compared to the protein groups above.

START

2.1 Account

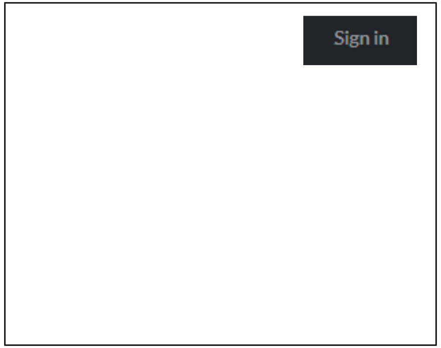

Figure 3.

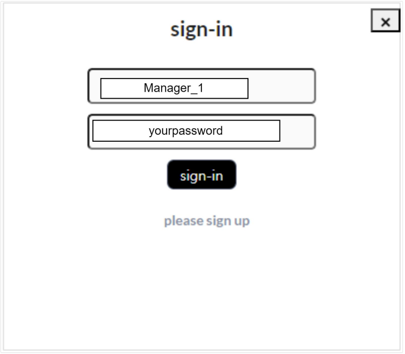

Figure 4

- Access ***http://riceproteome.plantprofile.net/***
- From here on, use the summarized screen to guide the explanation. Locate the 'Sign in' button (Figure 3).
- If it's your first visit, click the 'please sign up' button, enter an ID and password, and log in. In this manual, I have created 'Manager_1' to explain RiceProteomeDB (Figure 4).

2.2.1 Management(Create Project)

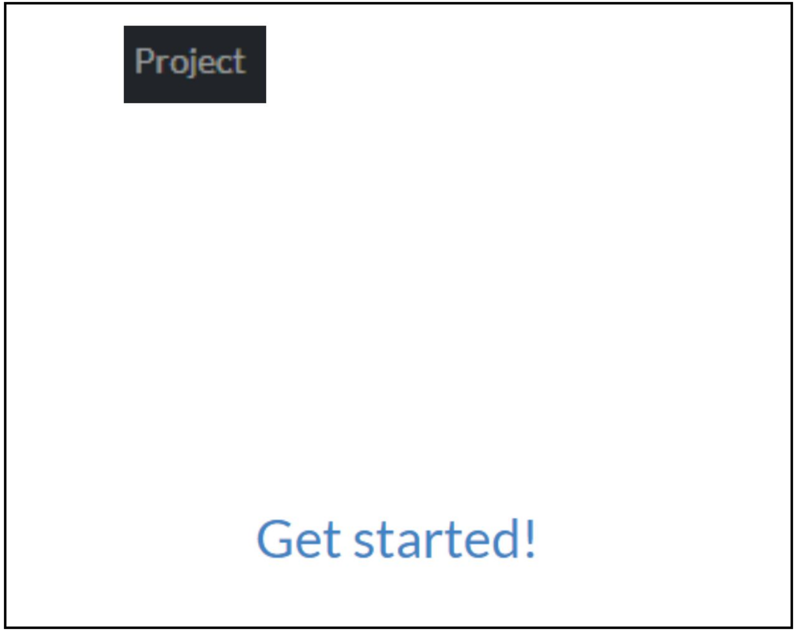

Figure 5

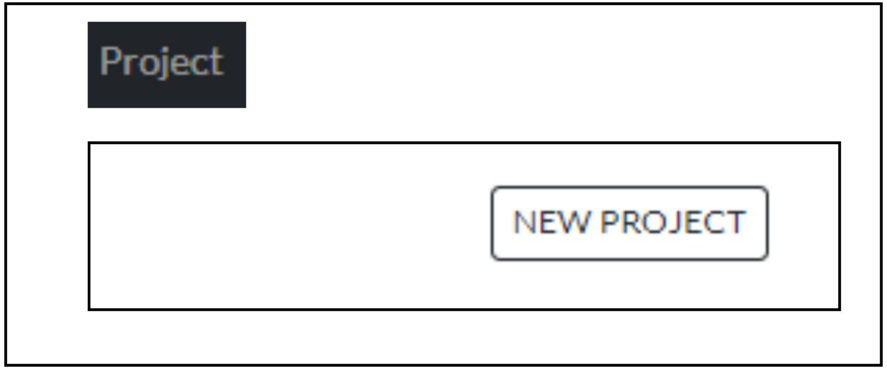

Figure 6

2.2.1 Management(Create Project)

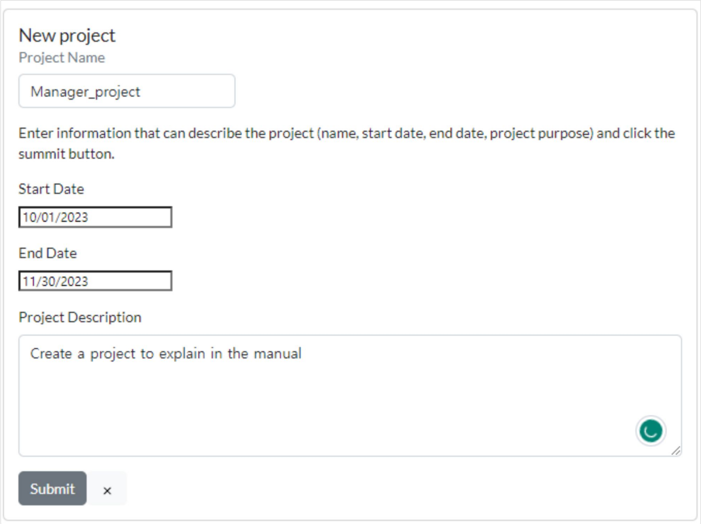

Figure 7

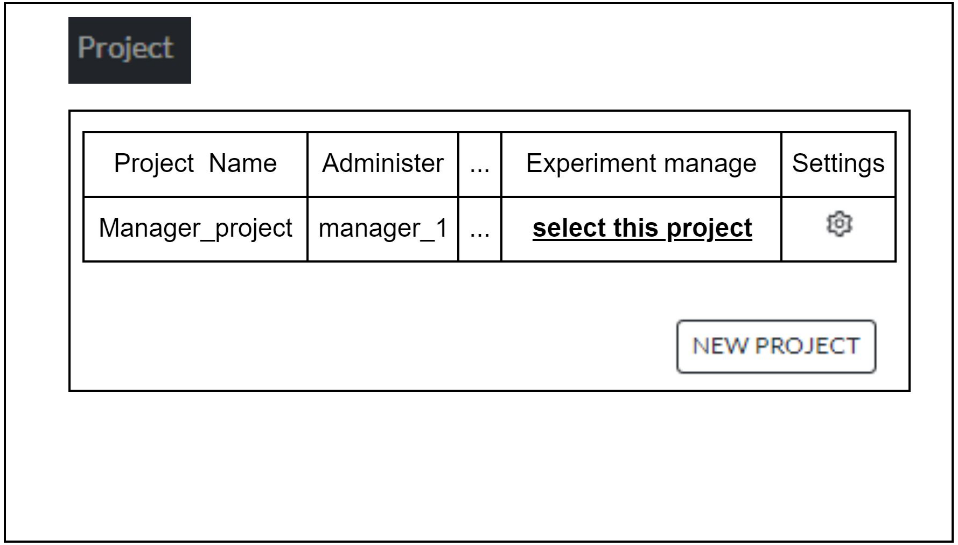

Figure 8

2.2.1 Management(Create Project)

- Find the 'Project' button(Figure 5).
- There is an empty space with a 'New PROJECT' button(Figure 6).
- Under 'New project', enter information including 'Project Name' as 'Manager_project'(Figure 7).
- As a result, you can see a table displaying project information including 'Project Name' as 'Manager_project', 'Administrator' as 'manager_1' an option to proceed to experiment management labeled 'select this project,' and the ability to add participants labeled 'Settings symbol’(Figure 8).

2.2.1.1 Management(Join Project)

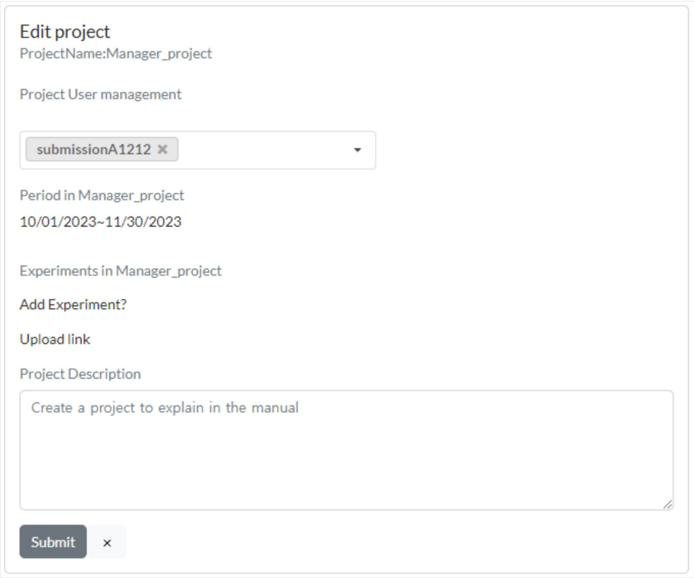

Figure 9

- You can add users to 'Manager_project' by clicking the 'Settings symbol.'
- As an example, I added 'submissionA1212.'
- Similar to the project creator 'manager_1,' 'submissionA1212' can upload experiments to the project(Figure 9).

2.2.2 Management(Upload Experiments)

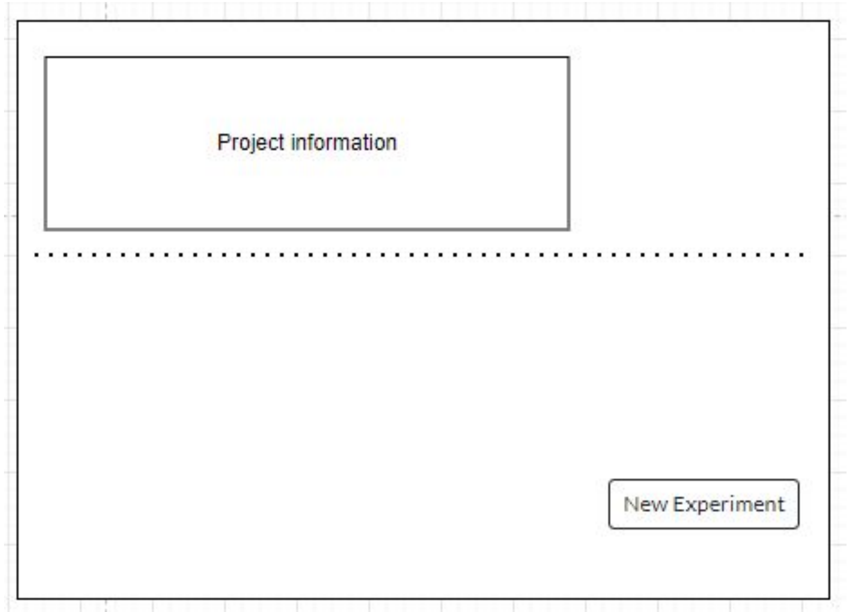

Figure 10

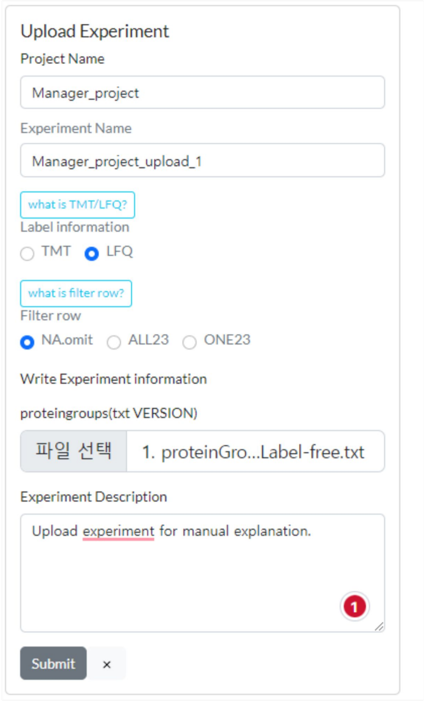

Figure 11

2.2.2 Management(Upload Experiments)

- When you select 'select this project' in 'Manager_project,' you can easily review the 'Project Information' section and the experiments added to the project. However, for first-time visitors, only the 'New Experiment' button is visible(Figure 10).
- I uploaded 'Proteingroups quantified by maxquant for label-free data' to demonstrate in the manual. The 'Experiment Name' was set as 'Manager_project_upload_1,' the 'Label Information' was 'LFQ,' and the 'Filter Row' was selected as 'NA.omit.' I provided an experiment description as 'upload experiment for manual explanation'(Figure 11).
- Add some sample information and additional descriptions at https://github.com/dongu7610/Riceproteome/wiki/RiceProteome_Manual-additional-explanation

2.2.2 Management(Upload Experiments)

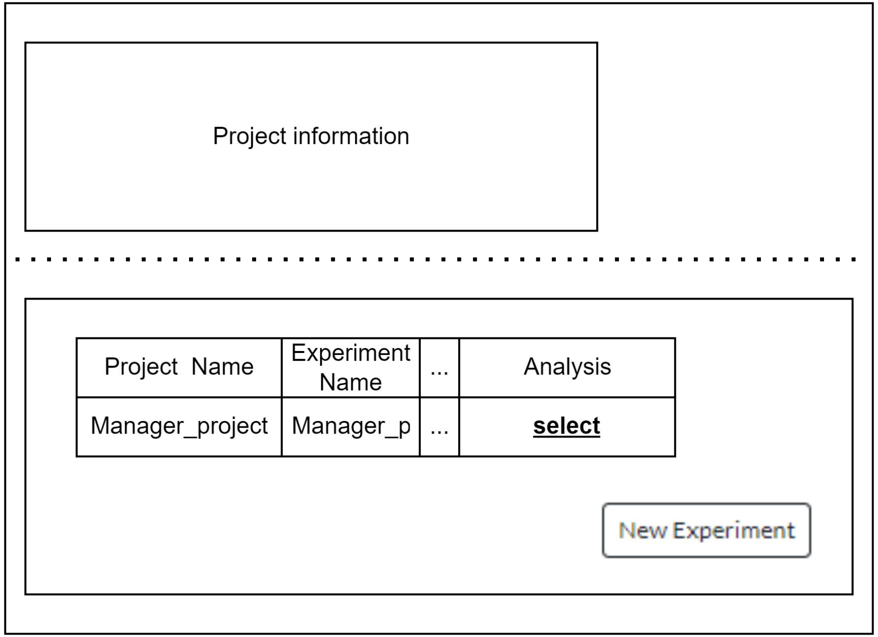

Figure 12

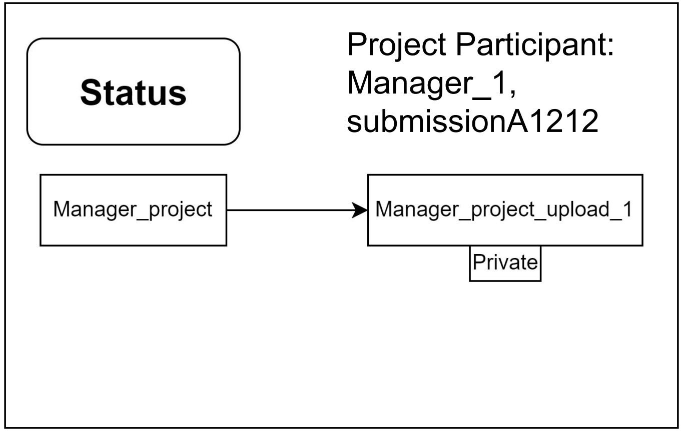

Figure 13

2.2.2 Management(Upload Experiments)

- Comparing to Figure 10, an entry labeled 'Experiment Name' as 'Manager_project_upload_1' has been added, which can be accessed for analysis via 'select'(Figure 12).
- To summarize the situation, the 'manager_1' account created a project named 'Manager_project,' involving the 'submissionA1212' account in the project. Additionally, 'Manager_project_upload_1' was uploaded for analysis. It is in a private state, thus not visible to other users(Figure 13).

2.2.3 Management(Add Analysis)

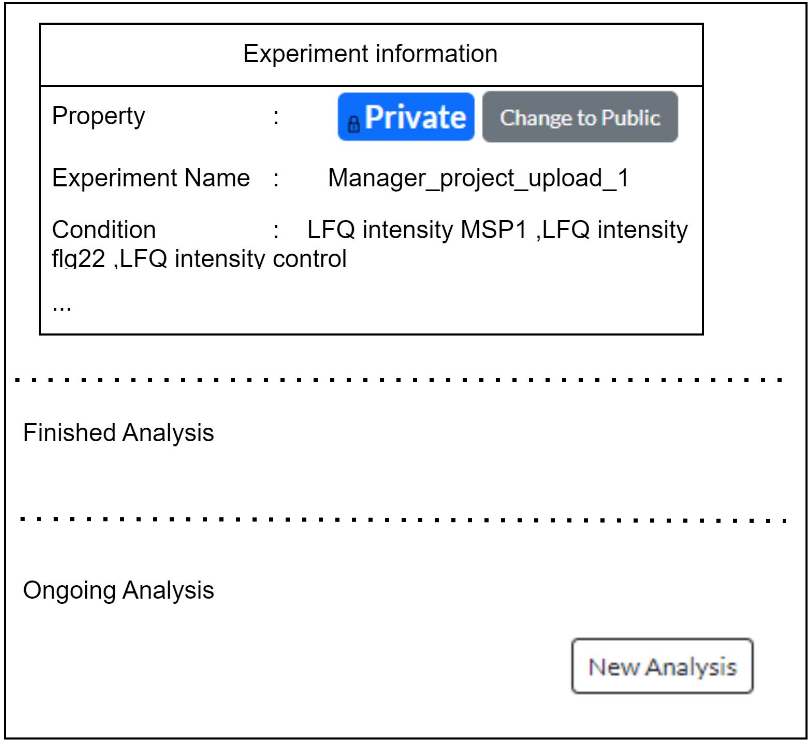

Figure 14

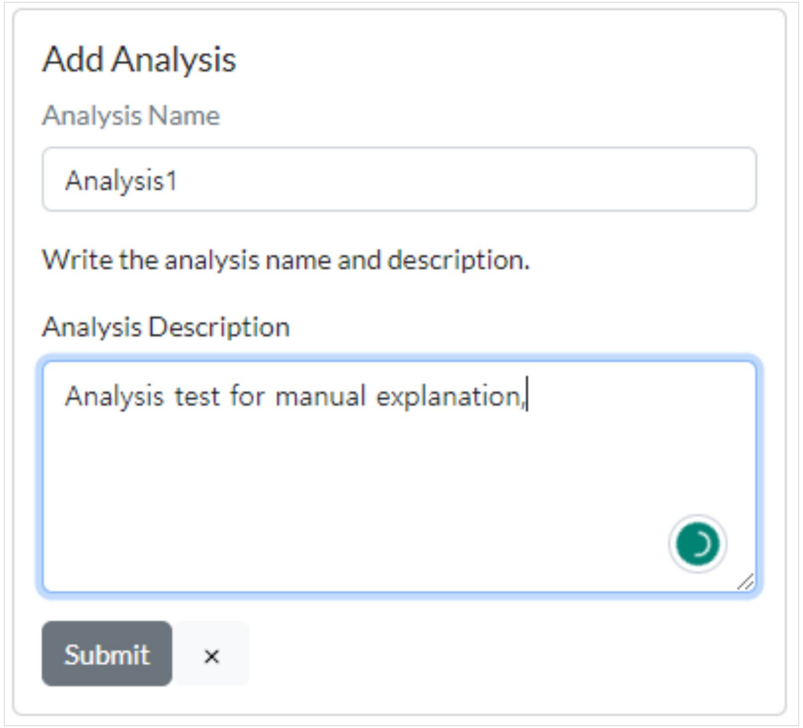

Figure 15

2.2.3 Management(Add Analysis)

- When accessing 'Manager_project_upload_1' via the 'select button,' it displays the 'Experiment Information,' including 'Experiment Name' and 'Condition.' Additionally, it features the 'Finished Analysis' section for completed analyses and the 'Ongoing Analysis' section for analyses in progress. However, for first-time visitors, only 'New Analysis' is visible(Figure 14).
- There is also a section where you can change the 'Private' to 'Public'.
- I created an 'analysis branch' where analysis can be conducted. In this manual, I selected the 'New Analysis' button and then entered 'Analysis1' for the 'Analysis Name' and 'Analysis test for manual explanation' for the 'Analysis Description'(Figure 15).

2.2.3 Management(Add Analysis)

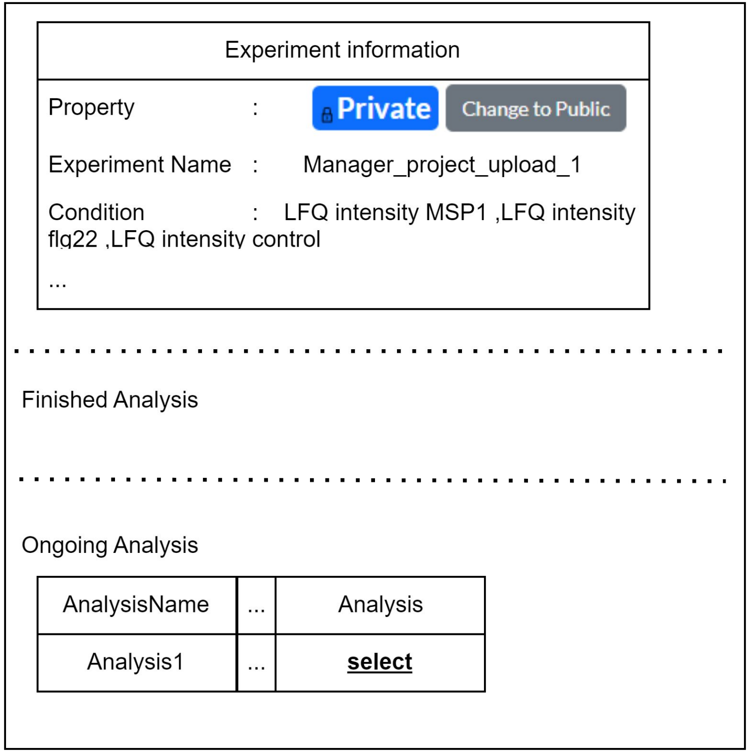

Figure 16

- Based on the input in 'Figure 15,' an entry 'Analysis1' appears under 'Ongoing Analysis,' accessible through the 'select button'(Figure 16).
- Once 'Analysis1' is completed, another analysis can be performed by using the 'New Analysis' button

2.3 Ongoing Analysis

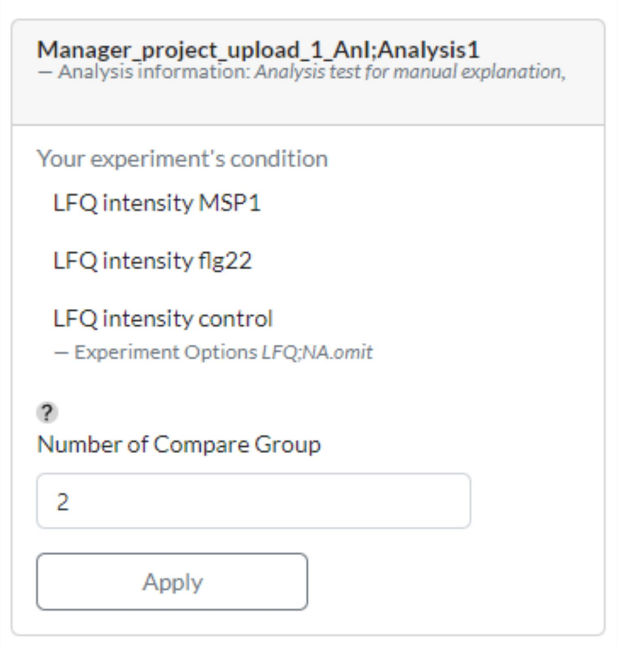

- When initiating the analysis via the 'select' button it displays the Analysis information along with Your experiment's condition. In this context, the experiment's conditions are LFQ intensity MSP1, LFQ intensity flg22, and LFQ intensity control
- Here, set the 'Number of Compare Groups' to 2 and click 'Apply' button(Figure 17).

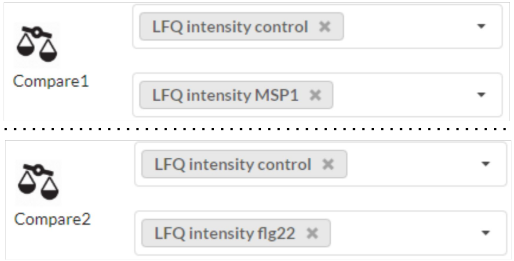

- 'compare 1' and 'compare 2,' and a dropdown menu appears.
- Enter 'control vs msp1' and 'control vs flg22' for each compare group(Figure 18).
- T-test analysis for each group.

Figure 17

Figure 18

2.3.1 Ongoing Analysis(Analysis DEP)

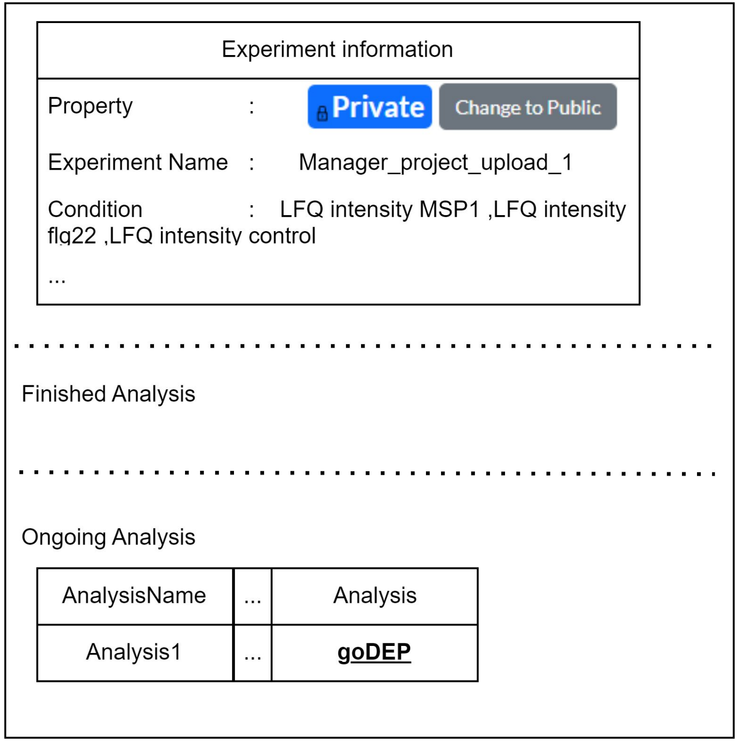

Figure 19

- After entering, wait for a moment; the 'select' will change to 'goDEP'(Figure 19).

2.3.1 Ongoing Analysis(Analysis DEP)

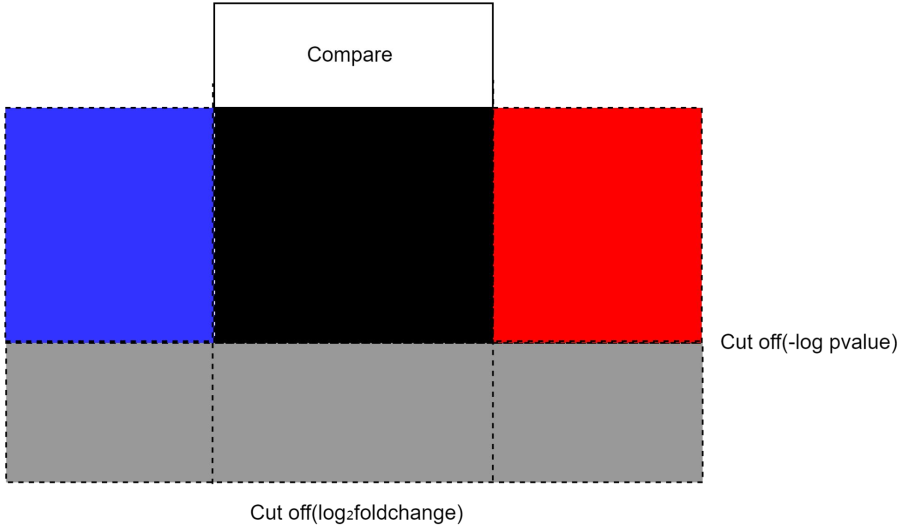

Figure 19

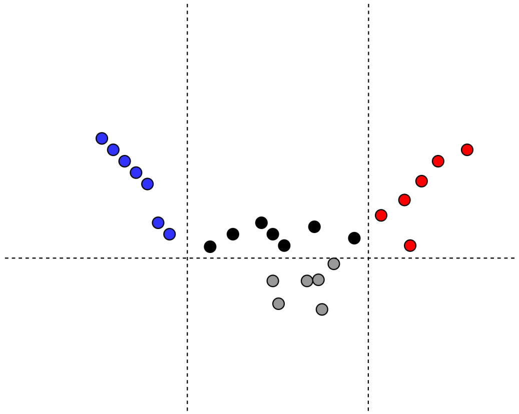

Figure 20

2.3.1 Ongoing Analysis(Analysis DEP)

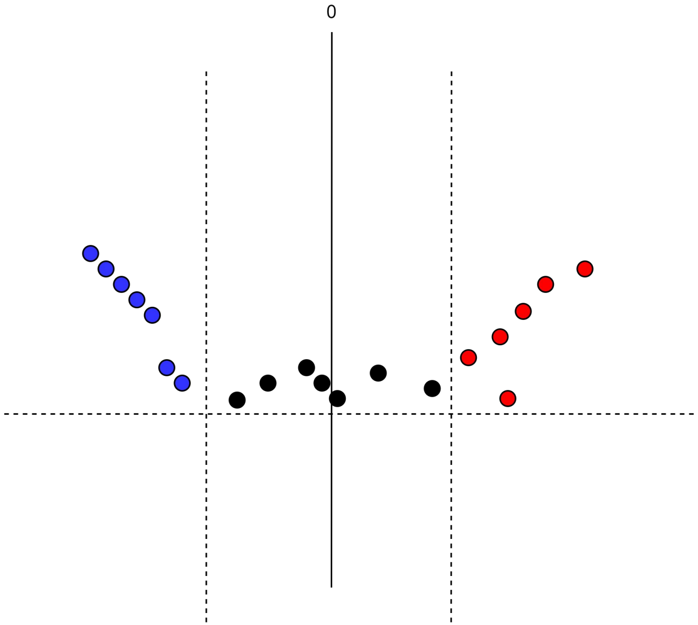

Figure 21

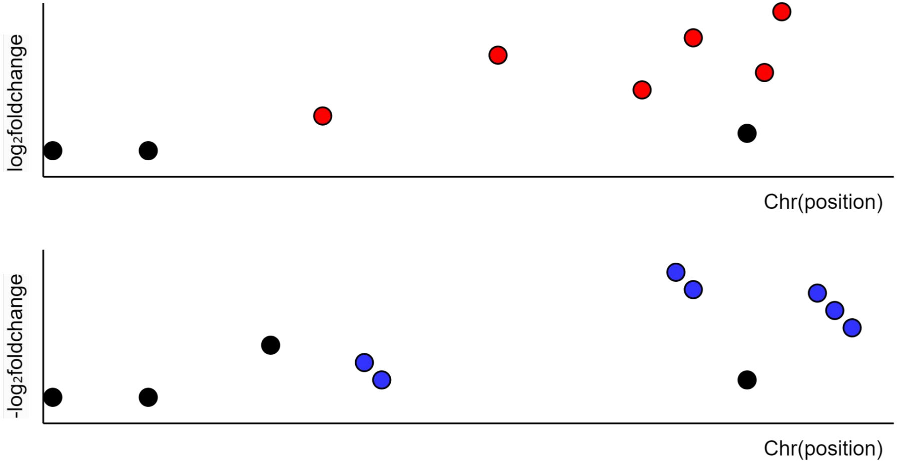

Figure 22

2.3.1 Ongoing Analysis(Analysis DEP)

- For the Volcano plot the x-axis represents 'log_2fold change,' and the y-axis represents '-log p-value.' The default 'cut-off' values are set at log_2fold change ‘-1.5, 1.5' and -log p-value 3. In this plot, the gray area signifies proteins eliminated based on p-value. The blue area represents down-regulated proteins in comparison, the black area designates similar proteins, and the red area signifies up-regulated proteins(Figure 19).
- Simply put, it can be graphed as follows(Figure 20).
- The Manhattan plot displays the log_2fold change values of proteins that have passed through the p-value, alongside their chromosome positions(Figure 21,Figure 22).

2.3.1 Ongoing Analysis(Analysis DEP)

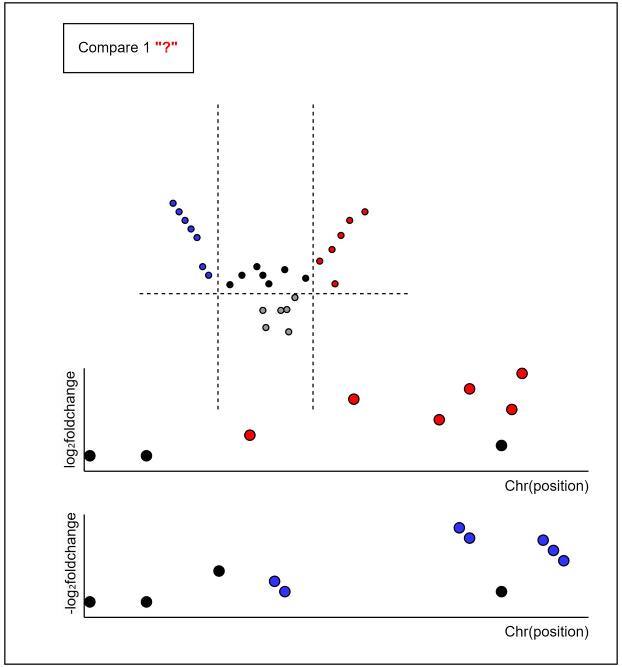

Figure 23

- In the manual, two comparisons, namely, compare1 and compare2, are prepared. A total of two volcano plots and four Manhattan plots are generated for each comparison(Figure 23).
- The red question mark indicates that clicking it allows you to verify which conditions were used.
- “compare 1(Control vs MSP1)”

2.3.1 Ongoing Analysis(Analysis DEP)

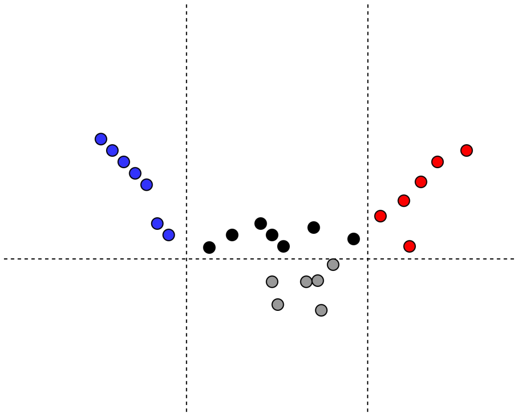

Figure 24

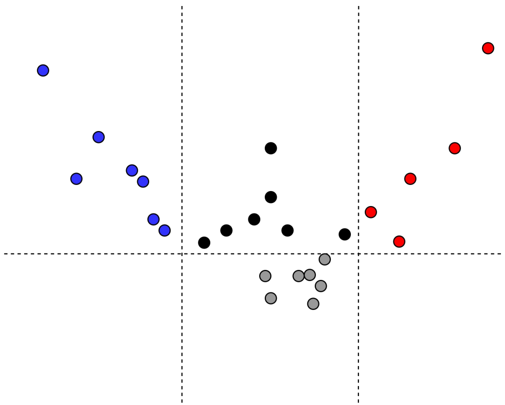

Figure 25

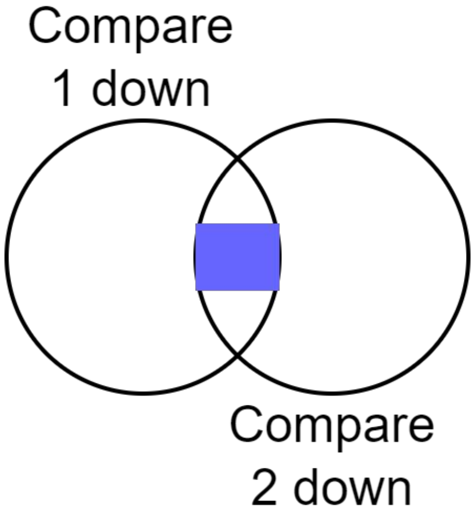

Figure 26

2.3.1 Ongoing Analysis(Analysis DEP)

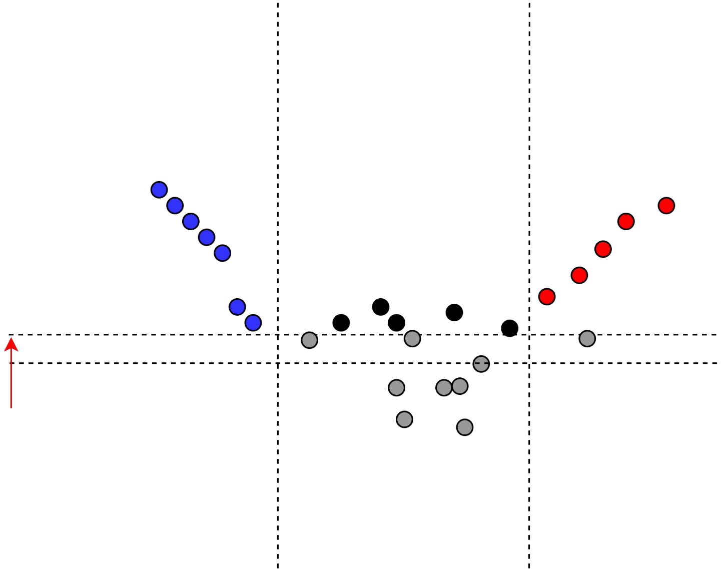

Figure 27

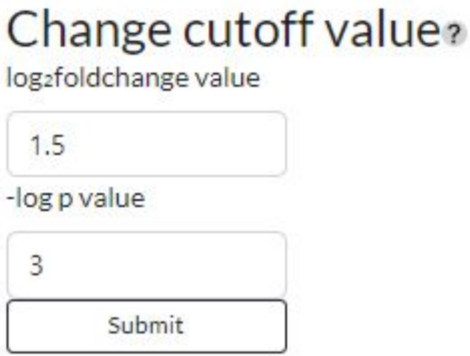

Figure 28

2.3.1 Ongoing Analysis(Analysis DEP)

- To prepare for network analysis, a Venn diagram is drawn for proteins that pass the cutoff p-value.
- For instance, there might be a different protein composition between compare 1 down and compare 2 down, but there could be an overlapping area(Figure 24,25).
- This overlap could be labeled as compare 1 down, compare 2 down for further analysis(Figure 26).
- You can tighten the cutoff to perform a more stringent analysis. By adjusting the p-value, represented by the red arrow, more proteins will be filtered out(Figure 27,28).

2.3.1 Ongoing Analysis(Analysis DEP)

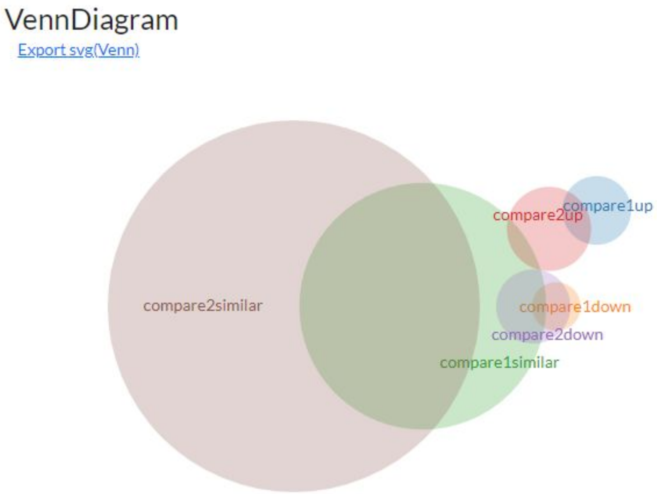

Figure 29

- This is the actual Venn diagram on the page. By clicking between compare1up and compare2up, it selects four proteins found in this region, and a Manhattan plot is drawn(Figure 29).

2.3.2 Ongoing Analysis(Analysis GO)

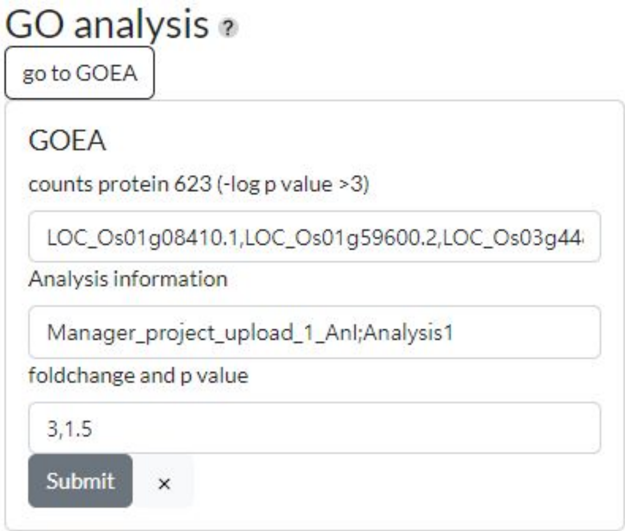

Figure 30

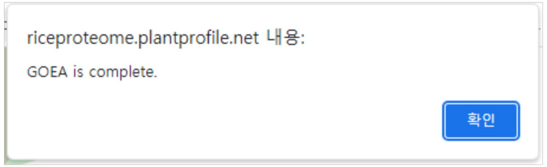

Figure 31

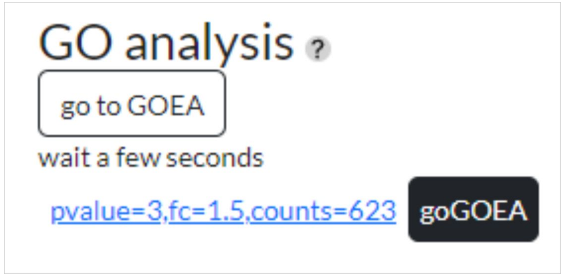

Figure 32

2.3.2 Ongoing Analysis(Analysis GO)

- I conduct GO analysis using these proteins that have passed the p-value threshold(Figure 30).
- After entering the details and waiting a few minutes, a completion window appears(Figure 31).
- Then, links and buttons for further progress are displayed(Figure 32).

2.3.2 Ongoing Analysis(Analysis GO)

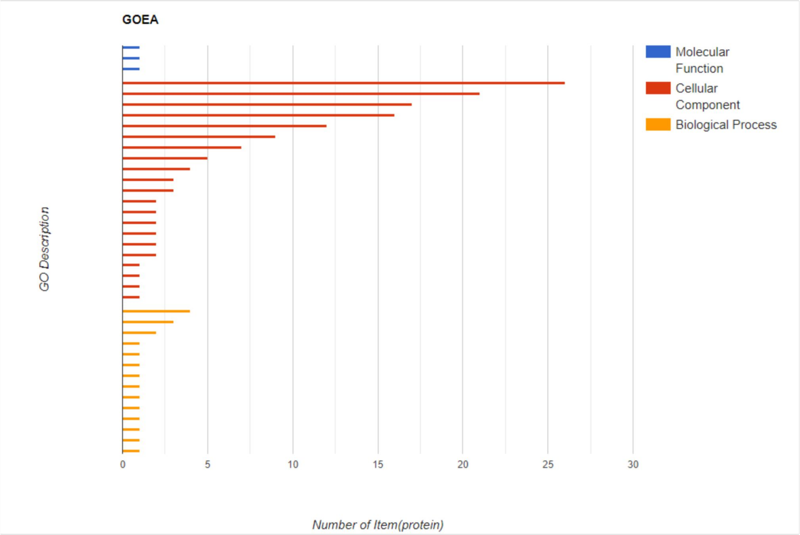

Figure 33

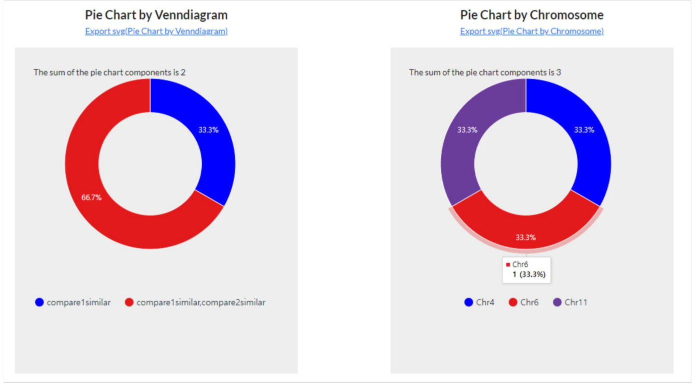

Figure 34

2.3.2 Ongoing Analysis(Analysis GO)

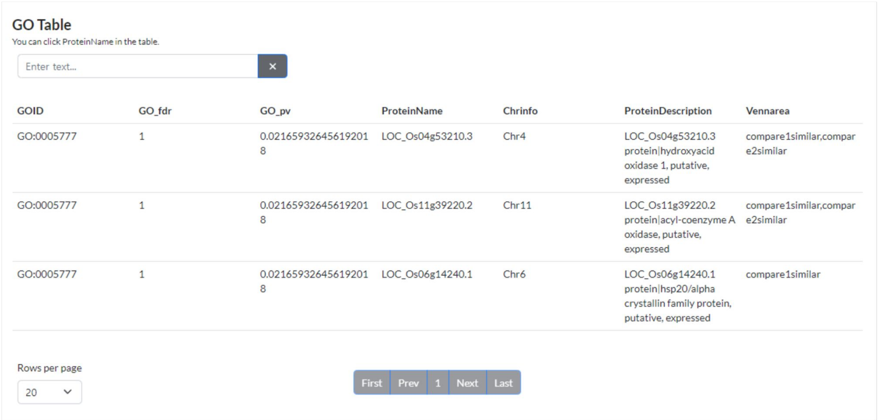

GO Table

You can click ProteinName in the table.

Enter text...

GOID	GO_fdr	GO_pv	ProteinName	Chrinfo	ProteinDescription	Vennarea
GO:0005777	1	0.021659326456192018	LOC_Os04g53210.3	Chr4	LOC_Os04g53210.3 protein|hydroxyacid oxidase 1, putative, expressed	compare1similar,compare2similar
GO:0005777	1	0.021659326456192018	LOC_Os11g39220.2	Chr11	LOC_Os11g39220.2 protein|acyl-coenzyme A oxidase, putative, expressed	compare1similar,compare2similar
GO:0005777	1	0.021659326456192018	LOC_Os06g14240.1	Chr6	LOC_Os06g14240.1 protein|hsp20/alpha crystallin family protein, putative, expressed	compare1similar

Rows per page 20

First Prev 1 Next Last

Figure 35

- This is the output from the Python library 'goatools.' From the results, I created a bar chart for three major categories(Figure 33).
- Among these, if you click the bar related to GO:0005777 (peroxisome), it displays a corresponding pie chart and table. In the Pie Chart by Venn diagram, the proteins connect with our experimental data. On the other hand, the Pie Chart by Chromosome connects protein information with their location data(Figure 34).
- The table provides information about the proteins associated with the selected GOID (GO:0005777)(Figure 35).

2.3.3 Ongoing Analysis(Protein basket)

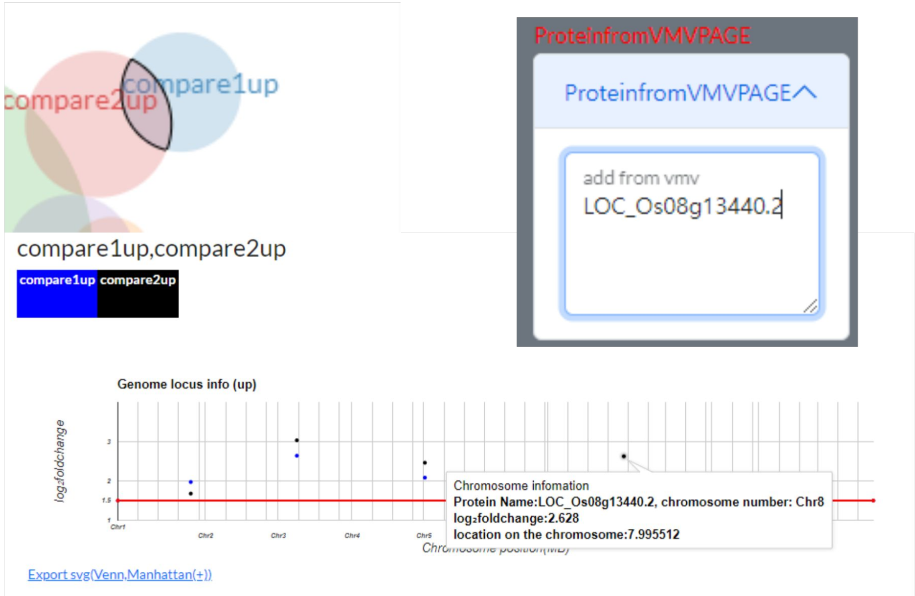

Figure 36

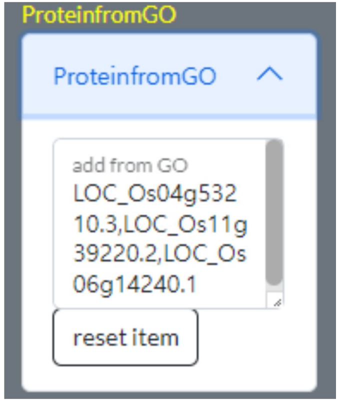

Figure 37

2.3.3 Ongoing Analysis(Protein basket)

- In RiceProteomeDB, you can add proteins you are interested in to the Protein Basket.
- For example, you can add the protein 'LOC_Os08g13440.2' among the proteins between 'compare1up' and 'compare2up' to the 'ProteinfromVMVPAGE'(Figure 36).
- Additionally, on the page performing the Analysis GO, by selecting an entry in the 'ProteinName' column from the Table that appears when clicking 'GO:0005777 (peroxisome),' you can add it to 'ProteinfromGO'(Figure 35,37).
- You can also utilize the list of proteins you already know by pasting it in bulk.
- I have added three proteins related to peroxisomes (LOC_Os04g53210.3, LOC_Os11g39220.2, LOC_Os06g14240.1) to ProteinfromGO in this manual.
- By clicking 'Find nodes' with the three proteins in the Protein Basket, you can perform Network Analysis!

2.3.4 Ongoing Analysis(Analysis Network)

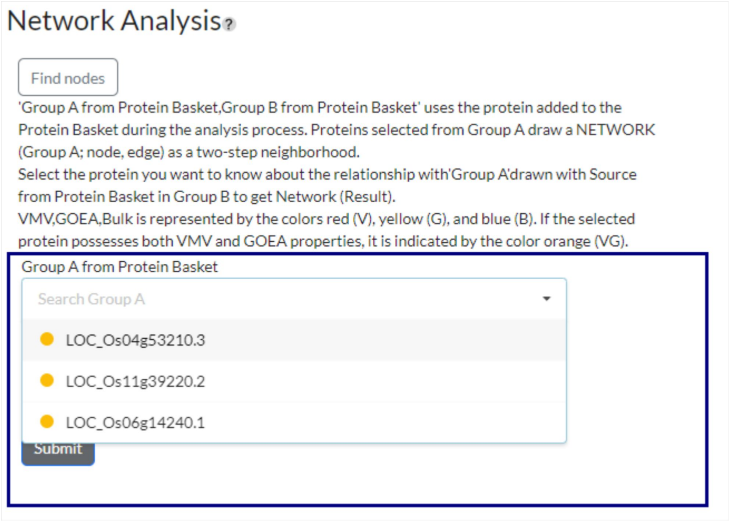

Figure 38

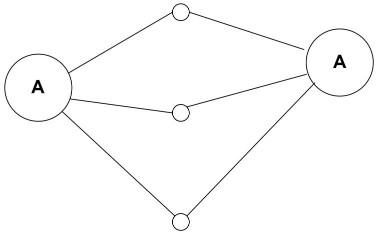

Figure 39

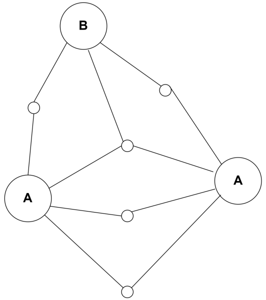

Figure 40

2.3.4 Ongoing Analysis(Analysis Network)

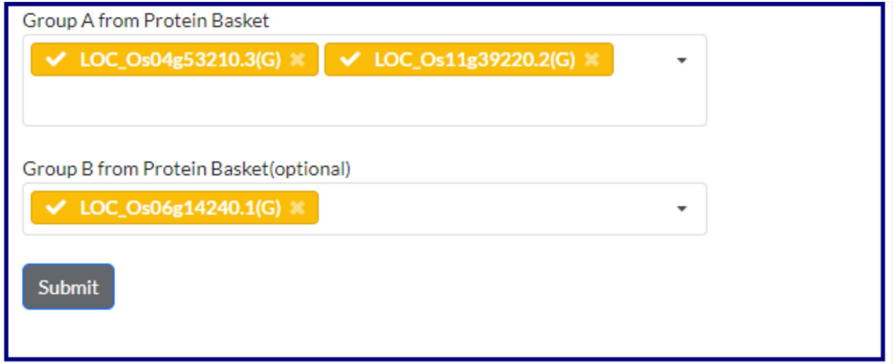

Figure 41

- Perform Network Analysis using the added proteins. The three proteins added from ProteinfromGO will be displayed(Figure 38).
- When choosing the nodes for the network, you select whether to draw the network as added nodes to Group A(Figure 39), or consider the options from Group B as well(Figure 40).
- In this manual, the analysis was conducted considering the Group B option(Figure 41).

2.3.4 Ongoing Analysis(Analysis Network)

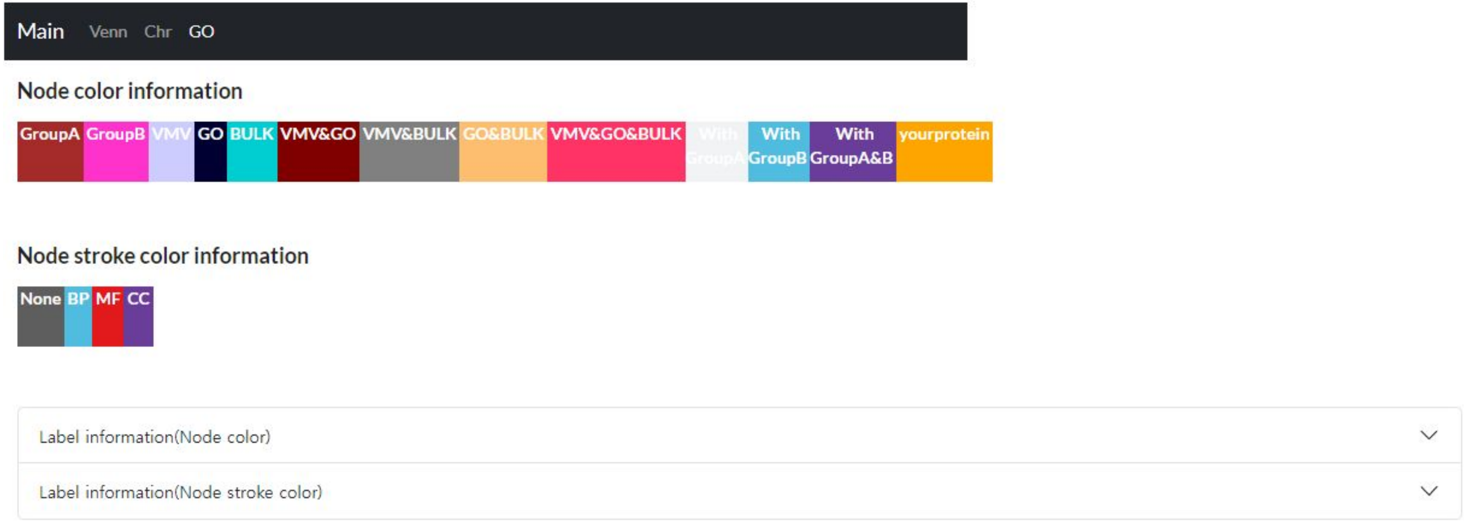

Figure 42

2.3.4 Ongoing Analysis(Analysis Network)

- From the results of the network analysis, there exists a Navigation bar comprising ‘Main’, ‘Venn’, ‘Chr’, ‘GO’ along with Node color and Node stroke color options.
- 'Main' represents the Normal Network. 'Venn' signifies the network with added experimental data.'Chr' provides Chromosome information, and 'GO' includes the BP, CC, MF categories.
- By expanding 'Label information' below (Node color, Node stroke color), you can easily review the simple Label information(Figure 42).

2.3.4 Ongoing Analysis(Analysis Network)

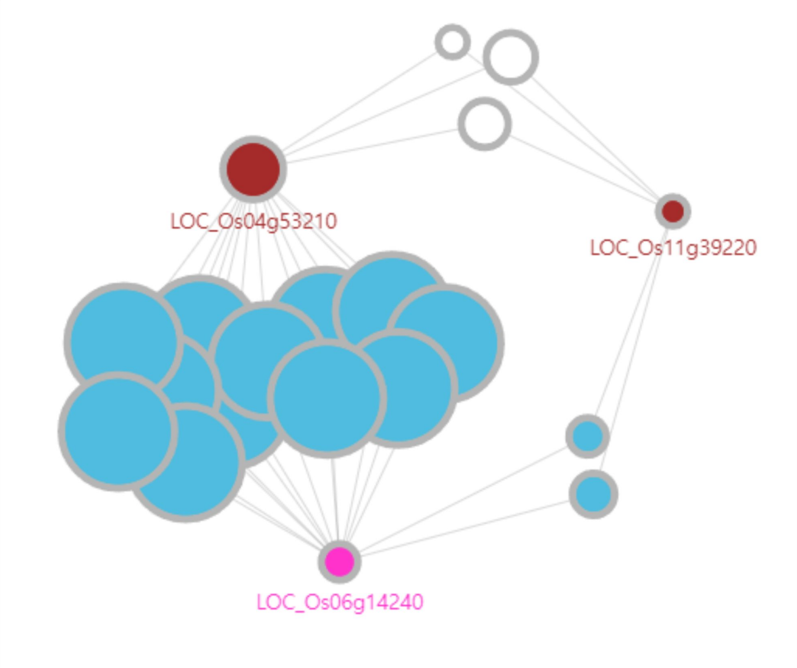

Figure 43(Main)

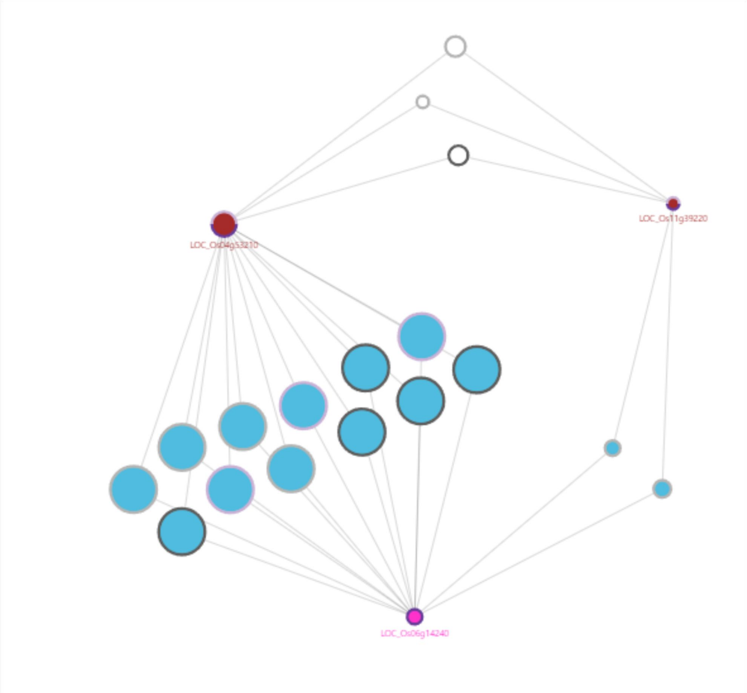

Figure 44(Venn)

2.3.4 Ongoing Analysis(Analysis Network)

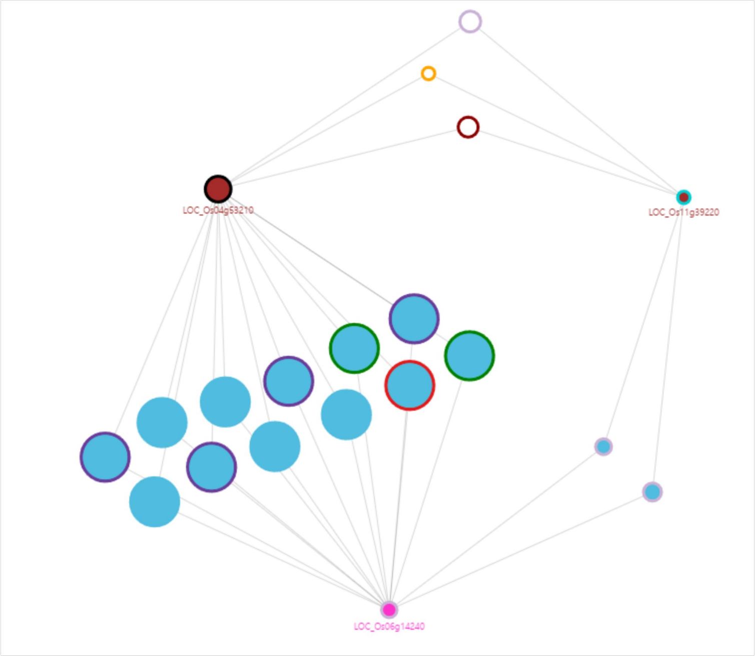

Figure 45(Chr)

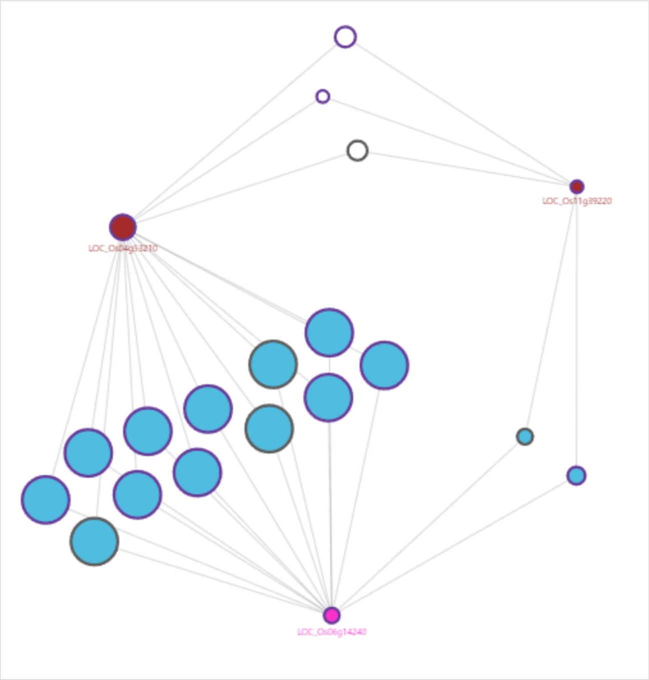

Figure 46(GO)

2.3.4 Ongoing Analysis(Analysis NETWORK)

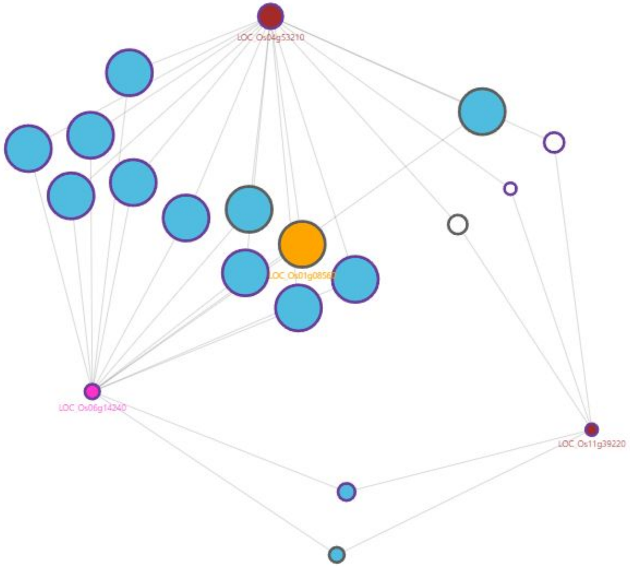

Figure 47

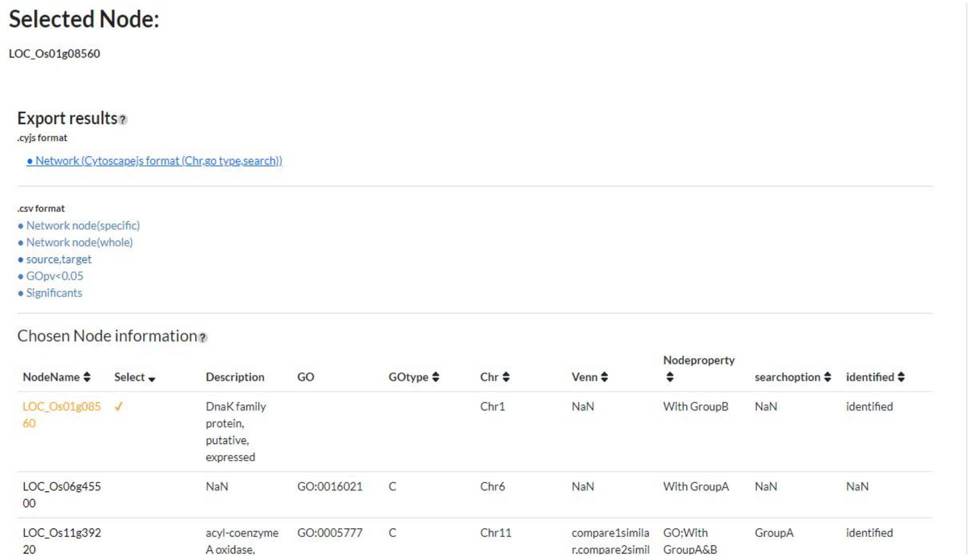

Selected Node:

LOC_Os01g08560

Export results?

.cyjs format

- Network (Cytoscapejs format (Chr,go type,search))

.csv format

- Network node(specific)
- Network node(whole)
- source,target
- GOpv<0.05
- Significants

Chosen Node information?

NodeName	Select	Description	GO	GOtype	Chr	Venn	Nodeproperty	searchoption	identified
LOC_Os01g08560	✓	DnaK family protein, putative, expressed			Chr1	NaN	With GroupB	NaN	identified
LOC_Os06g45500		NaN	GO:0016021	C	Chr6	NaN	With GroupA	NaN	NaN
LOC_Os11g39220		acyl-coenzyme A oxidase,	GO:0005777	C	Chr11	compare1similar,compare2simil	GO;With GroupA&B	GroupA	identified

Figure 48

2.3.4 Ongoing Analysis(Analysis NETWORK)

- The Network in 'Figure(43-46)' represents individual examinations of 'Main,' 'Venn,' 'Chr,' 'GO.' You can observe the change in Node Stroke colors.
- When a researcher discovers an interesting protein while examining, a left-click selection marks it in orange color.
- The selected protein is then added to the 'Selected Node.'
- In this manual, I chose LOC_Os01g08560(Figure 47) and was able to gather basic protein information from the row marked with a check in the Table(Figure 48).

2.4 Finish Analysis

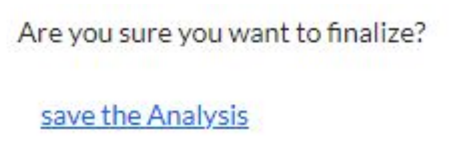

Figure 49

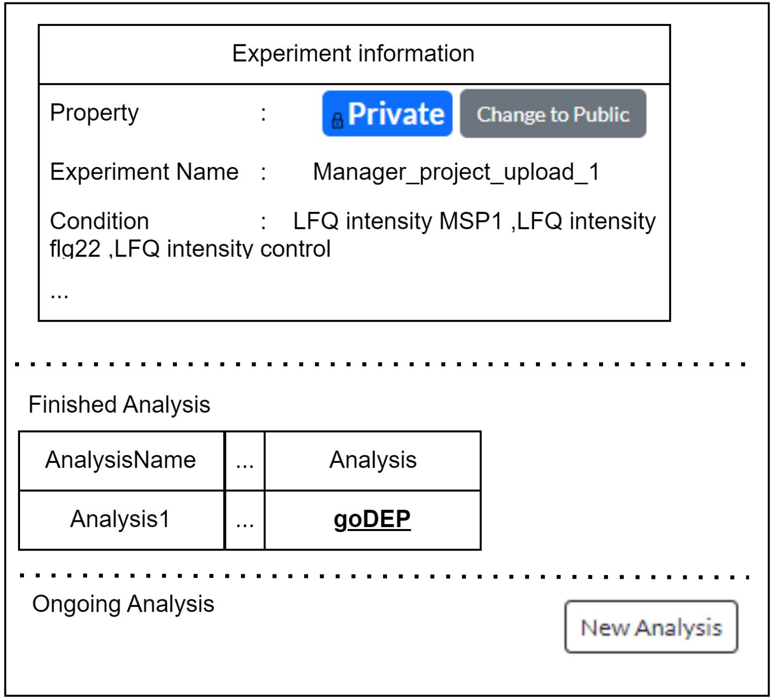

Figure 50

2.4 Finish Analysis

- When finishing the analysis, click on 'Save the Analysis' at the bottom(Figure 49).
- This saves the analysis, and 'Analysis1,' the analysis performed in this manual, is added to the 'Finished Analysis' section. Since one analysis is completed, you can proceed to add a new analysis(Figure 50).

CONTACT

- Further details not covered in this manual are explained on the GitHub Wiki page: ***https://github.com/dongu7610/Riceproteome/wiki/RiceProteome_Manual-additional-explanation*** For any additional queries or information not addressed in this manual, please refer to the GitHub Wiki.
- For any inquiries or additional information not covered in this manual, please contact us via email at ***dongu7610@naver.com***
